# Concurrent fibrinogen Aα-chain amyloidosis and AA amyloidosis in a Humboldt penguin (*Spheniscus humboldti*)

**DOI:** 10.1007/s11259-026-11572-y

**Published:** 2026-10-10

**Authors:** Susumu Iwaide, Natsumi Kobayashi, Mako Ikudome, Shiho Takezawa, Yoshiyuki Itoh, Tomoyuki Shibahara, Keisuke Kawase, Tomoaki Murakami

**Affiliations:** 1 Research Unit of Innovative One Medicine, Advanced Research Center for One Welfare, Tokyo University of Agriculture and Technology, 3-5-8 Saiwai- cho, Fuchu, Tokyo, Japan; 2 Laboratory of Veterinary Toxicology, Tokyo University of Agriculture and Technology, 3-5-8 Saiwai-cho, Fuchu, Fuchu, Tokyo, Japan; 3 National Institute of Animal Health, National Agriculture and Food Research Organization, 3-1-5 Kannondai, Tsukuba, Ibaraki Japan; 4 Ibaraki Prefectural Kenpoku Livestock Hygiene Service Center, 966-1 Nakagachi-cho, Mito, Ibaraki Japan; 5 Smart-Core-Facility Promotion Organization, Tokyo University of Agriculture and Technology, 3-5-8 Saiwai-cho, Fuchu, Tokyo, Japan; 6 Department of Veterinary Immunology, Graduate School of Veterinary Medical Sciences, Osaka Metropolitan University, 1-58 Rinku- oraikita, Izumisano, Osaka Japan; 7 Hitachi Kamine Zoo, 5-2-22, Miyata-cho, Hitachi, Ibaraki Japan

**Keywords:** Avian, Double amyloidosis, Dual amyloidosis, Penguin, Serum amyloid A

## Abstract

Amyloidosis is a disease characterized by the deposition of abnormally folded proteins as amyloid fibrils in tissues and occurs in various mammals and birds. In some cases, amyloids derived from two different precursor proteins may coexist in the same individual, a condition known as double amyloidosis. Amyloid A (AA) amyloidosis is known to occur frequently in birds; however, little is known about other types of amyloidosis. In the present study, we report a case of amyloidosis in a Humboldt penguin involving two distinct types of amyloids. Histopathological examination, transmission electron microscopy, mass spectrometry-based proteomic analysis, and immunohistochemistry revealed the concurrent occurrence of fibrinogen Aα-chain (FibA) amyloidosis and AA amyloidosis. FibA-derived and AA amyloid deposits exhibited distinct nodular and diffuse morphologies, respectively, suggesting that their deposition patterns may provide a morphological clue for distinguishing between the two types of amyloids. To our knowledge, this is the first case of FibA amyloidosis in a penguin and the first report of the concurrent occurrence of two distinct types of systemic amyloidosis in a bird.

**Supplementary Information:**

The online version contains supplementary material available at https://doi.org/10.1007/s11259-026-11572-y.

## Background

Amyloidosis is a group of diseases characterized by the deposition of misfolded protein (amyloid); approximately 42 types are known in humans and about 20 in animals, depending on the type of precursor proteins (Buxbaum et al. 2024; Iwaide et al. 2025a). Amyloidosis is classified into systemic and localized forms, depending on whether amyloid deposits occur at sites distant from or close to the site of precursor protein production (Buxbaum et al. 2024). Amyloidosis is also commonly diagnosed in birds. Amyloid A (AA) amyloidosis, which is derived from serum amyloid A (SAA) and associated with chronic inflammation, is the most common form of systemic amyloidosis in birds (Iwaide et al. 2025a). As for amyloidosis subtypes other than AA in birds, systemic fibrinogen Aα-chain (FibA) amyloidosis has been reported in a blue-winged kookaburra, and cerebral amyloid β (Aβ) amyloidosis has been reported in a flamingo, a great spotted woodpecker, and three eagles (Nakayama et al. 1999; Kimoto et al. 2015; Ono et al. 2020; Murakami et al. 2026). Systemic amyloidosis has also been described in an African penguin (*Spheniscus demersus*) (Kim et al. 2022); however, information on amyloid types and potential predisposing factors in penguins remains limited.

Double amyloidosis is a condition in which amyloid derived from two different proteins is deposited simultaneously in the same individual, and it occurs sporadically in humans (Dalland et al. 2025). In contrast, information on the concurrent deposition of multiple amyloid types in animals remains limited, although cases involving multiple distinct amyloid types have been reported (Nakamura et al. 2008; Iwaide et al. 2026). In birds, co-occurrence of cerebral Aβ amyloidosis and systemic AA amyloidosis have been reported in a flamingo (Ono et al. 2020). However, to our knowledge, the simultaneous occurrence of two distinct types of systemic amyloidosis in a single individual has not been reported in birds.

FibA amyloidosis is a disease characterized by FibA-derived amyloid deposition (Yazaki et al. 2018). Among mammals, it has been reported not only in humans but also in Japanese squirrels, dogs and Japanese raccoon dogs (Yazaki et al. 2018; Iwaide et al. 2023a, 2025b; Kobayashi et al. 2026; Yoshikura et al. 2026). In avians, however, it has only been reported in a blue-winged kookaburra (*Dacelo leachii*), and due to the small number of cases and other factors, its precise pathogenesis remains unclear (Murakami et al. 2026). In this study, we describe a case of double amyloidosis involving FibA amyloidosis and AA amyloidosis in a Humboldt penguin.

## Case presentation

A 31-year-old male Humboldt penguin (*Spheniscus humboldti*) housed with five other conspecifics in captivity developed anorexia and died nine days later despite assisted feeding and subcutaneous fluid therapy. The bird had exhibited lameness for approximately six months during the year preceding its death. Arthritis or gout was clinically suspected as the cause of the lameness, and meloxicam had been administered as needed. A necropsy was performed to determine the cause of death. The bird weighed 2 kg at the time of the necropsy. During the necropsy, white nodules were detected in the coelomic serosa and the air sacs.

**Fig. 1:**
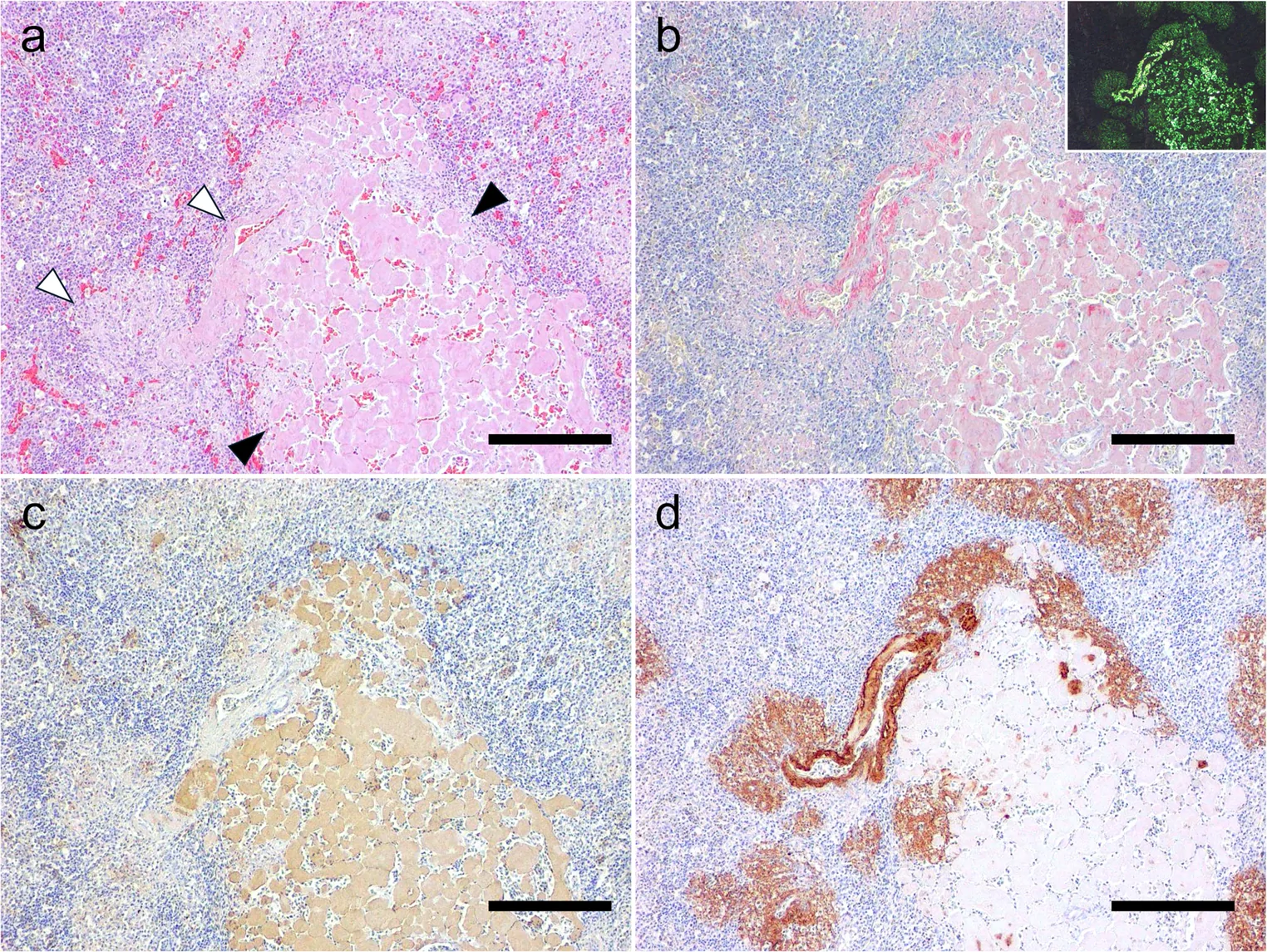
Amyloidosis, spleen, Humboldt penguin. Nodular amyloid deposits are present in the vascular walls (**a**, black arrowheads). In addition, diffuse amyloid deposits are present in the vascular walls and red pulp (**a**, white arrowheads). Both types of amyloid deposits stain pink-red (**b**) in Congo red and show yellow-green birefringence under polarized light (**b**, inset). Nodular and diffuse amyloid deposits are specifically immunolabeled with anti-fibrinogen Aα-chain (FibA) antibody (**c**) and anti-serum amyloid A (SAA) antibody (**d**), respectively. Immunohistochemistry for FibA (**c**) and SAA (**d**). Bars = 200 μm

The liver, spleen, kidneys, heart, lungs, small intestine, large intestine, and air sacs were sampled, fixed in 10% neutral buffered formalin, embedded in paraffin, and sectioned into 3 μm slices. Sections were stained with hematoxylin and eosin, and additional sections were subjected to Congo red and periodic acid-Schiff (PAS) reaction. Histopathological examination revealed extensive coagulative necrosis in the white nodules of the air sacs. Within these areas, many fungal hyphae were observed, and in the surrounding tissue, an inflammatory infiltrate consisting primarily of heterophils, multinucleated giant cells, and fibrin deposits was noted (Supplementary Fig. 1a and 1b). The hyphae were stained with PAS reaction (Supplementary Fig. 1c). Lymphocytes and plasma cells were distributed along the hepatic sinusoids, together with pigment-laden Kupffer cells. In the kidneys, calcifications were present in the walls of the renal tubules. In the lungs, multifocal calcifications were present in the interstitium and vascular walls. Mild heterophilic infiltration was present in the lamina propria and in the vascular walls of the intestine. Furthermore, eosinophilic, amorphous deposits were observed in multiple organs and exhibited two distinct morphological patterns: diffuse and nodular. In the liver, abundant, diffuse deposition was present in the perisinusoidal space. In contrast, moderate, nodular to spheroidal deposition was present around the vascular walls (Supplementary Fig. 2). In the spleen, abundant, diffuse deposition was present in the vascular walls and in the red pulp, whereas abundant, nodular deposits were present perivascularly in the white pulp (Fig. 1). In the kidneys, moderate, diffuse deposition was present in the interstitium and in the vascular walls (Supplementary Fig. 3). In the heart, moderate, diffuse deposition was observed in the myocardial interstitium and vascular walls. Additionally, moderate, nodular deposition was observed around vessels (Supplementary Fig. 4). In the lung, only a few small nodular deposits were present in the interstitium. In the esophagus and intestines, moderate, diffuse amyloid deposition was present in the lamina propria (Supplementary Fig. 5). Mild, diffuse amyloid deposition was present in the submucosa of the trachea. The material stained pink-red with Congo red and exhibited typical birefringence for amyloid under polarized light (Fig. 1b and Supplementary Figs. 2–5). Furthermore, the nodular amyloid deposits present in the liver, spleen, and heart were positive for the PAS reaction, but negative for Alcian blue at both pH 1.0 and 2.5 (Supplementary Fig. 6).

To examine the hyphae observed in the air sac, immunohistochemistry (IHC) was performed using an anti-*Aspergillus* spp. antibody (Clone: Mab-WF-AF-1: Dako, Glostrup, Denmark). Antigen retrieval was conducted using 0.1% actinase E solution in phosphate-buffered saline at 37 °C for 20 min. The sections were incubated with the primary antibody for 30 min at room temperature (approximately 24.5 °C). Histofine Simple Stain MAX-PO (MULTI) (Nichirei, Tokyo, Japan) and Simple Stain AEC Solution (Nichirei) were used as the secondary antibody and chromogenic substrate, respectively. As a result, the hyphae showed strong immunolabeling for *Aspergillus* spp. (Supplementary Fig. 1d).

**Fig. 2:**
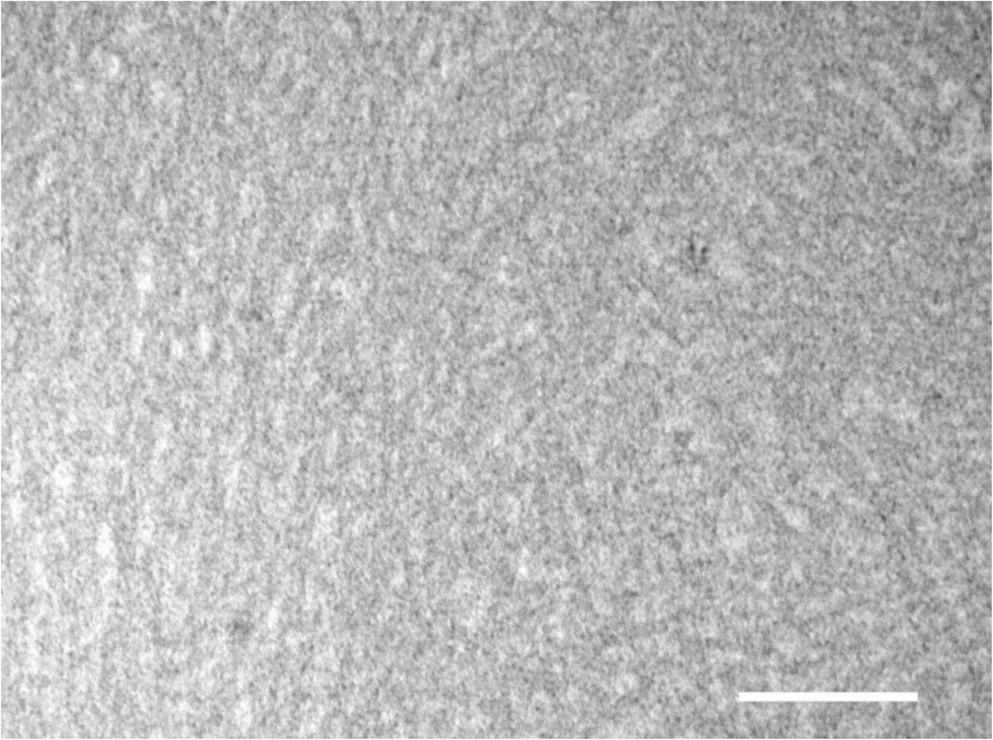
Amyloidosis, spleen, Humboldt penguin. The nodular amyloid deposits contain straight and unbranched fibrils with diameters of 13 nm that are arranged in dense bundles. Transmission electron microscopy. Bar = 200 nm

Transmission electron microscopy (TEM) was performed to visualize amyloid fibrils. Samples were prepared from nodular amyloid deposits of formalin-fixed, paraffin-embedded spleen section as previously described (Iwaide et al. 2025b), and observed using a transmission electron microscope (JEM-1400 Flash; JEOL, Tokyo, Japan). TEM examination of the nodular amyloid deposits revealed densely packed bundles of straight, unbranched fibrils approximately 13 nm in diameter (Fig. 2). TEM examination was not performed on the diffuse amyloid deposits.

To describe the proteome of amyloid deposits, we performed proteomic analysis using liquid chromatography-tandem mass spectrometry (LC-MS/MS). For formalin-fixed, paraffin-embedded samples of liver, spleen, and heart, 7-µm sections were cut onto polyethylene naphthalate slides (Thermo Fisher Scientific, Waltham, MA, USA) and stained with Congo red. Amyloid-containing tissue areas were manually dissected from Congo red-stained sections using 30-gauge needles (Becton, Dickinson and Co., Franklin Lakes, NJ, USA) under a stereomicroscope (SMZ745; Nikon, Tokyo, Japan), with dissected tissue areas ranging from 120,000 to 700,000 μm². From the liver, one sample of hepatic parenchyma containing diffuse amyloid deposits in the perisinusoidal spaces and one sample consisting exclusively of nodular amyloid deposits were collected. One sample each of nodular and diffuse vascular amyloid deposits was collected from both the spleen and the heart. In each organ, nodular and diffuse amyloid deposits were sampled separately to ensure that both types of deposits were not included in the same sample. LC-MS/MS analysis was performed according to previously reported methods (Iwaide et al. 2025b). The obtained LC-MS/MS data were searched against the predicted protein sequences of *Spheniscus humboldti* from the National Center for Biotechnology Information (NCBI) genome assembly (GCA_027474245) using Mascot Server (Matrix Science, London, UK), with trypsin specified as the proteolytic enzyme. To investigate the possibility of chemical modifications, an error-tolerant search was first performed on the data; subsequently, methionine oxidation (which was significantly detected) was used as an in silico variable modification. Among the detected peptides, those with p-values less than 0.05 were considered statistically significant and are presented in the results. As a result, SAA was detected as the major amyloid component in tissue samples of liver, spleen, and heart with diffuse amyloid deposits (Table 1), while FibA was detected in those with nodular amyloid deposits (Supplementary Table 1).

**Table 1:** Representative proteins detected by LC-MS/MS of amyloid deposits

Amyloid morphology		Accession	Description	Score	Sequences	emPAI*
Nodular amyloid deposits	Liver	KAM6266820**	Fibrinogen Aα-chain	471	15	0.25
		KAF1426833.1	Vitronectin, partial	310	19	0.55
		KAF1429209.1	Apolipoprotein A-I	19	1	0.11
	Spleen	KAM6266820**	Fibrinogen Aα-chain	1469	52	0.69
		KAF1426833.1	Vitronectin, partial	882	42	0.99
		KAF1429209.1	Apolipoprotein A-I	87	1	0.11
		KAF1429220.1	Apolipoprotein A-IV	78	4	0.27
	Heart	KAM6266820**	Fibrinogen Aα-chain	563	20	0.25
		KAF1426833.1	Vitronectin, partial	175	10	0.28
Diffuse amyloid deposits	Liver	KAF1429209.1	Apolipoprotein A-I	82	3	0.38
		KAF1408665.1	Serum amyloid A protein	55	2	0.24
	Spleen	KAF1426833.1	Vitronectin, partial	17	1	0.06
		KAF1408665.1	Serum amyloid A protein	73	2	0.54
	Heart	KAF1408665.1	Serum amyloid A protein	363	13	1.37
		KAF1426833.1	Vitronectin, partial	171	9	0.37
		KAF1429209.1	Apolipoprotein A-I	30	1	0.11
		KAF1410998.1	Collagen alpha-1(VI) chain	29	1	0.15

* emPAI, exponentially modified protein abundance index

** The amino acid sequence used for the database search was independently predicted from the Humboldt penguin genome and was identical to the subsequently deposited sequence indicated by the accession number

The FibA sequence of Humboldt penguin listed in the genome assembly was incomplete, and the full-length protein sequence had not been predicted (NCBI accession: KAF1405730). Therefore, using BLAST (https://blast.ncbi.nlm.nih.gov/Blast.cgi) and the annotated FibA sequence of the closely related emperor penguin (*Aptenodytes forsteri*; XP_009281595) as the reference, we predicted the complete *FGA* open reading frame from the Humboldt penguin reference genome and translated it into the corresponding amino acid sequence. The FibA sequence obtained using this method was 100% identical to the Humboldt penguin FibA sequence that was subsequently deposited in the NCBI database (NCBI accession: KAM6266820). After incorporating the predicted Humboldt penguin FibA sequence into the Mascot search database and repeating the Mascot analysis, the number of FibA-derived peptides increased, and FibA was the most frequently identified protein in the nodular amyloid deposits of the liver, spleen, and heart (Table 1 and Supplementary Table 2). Either SAA or FibA was detected in each of the analyzed amyloid deposits, and the two precursor proteins were never detected together within the same deposit. Furthermore, no peptides derived from other previously reported amyloid precursor proteins were detected. Vitronectin, apolipoprotein A-I, apolipoprotein A-IV and collagen alpha-1 (VI) chain were detected as amyloid signature proteins in some amyloid deposits (Palstrøm et al. 2021; Iwaide et al. 2025a).

To identify the amyloid precursor proteins, we performed IHC using in-house anti-FibA and anti-SAA (Iwaide et al. 2023b) antibodies. An anti-FibA antibody was generated using custom antibody production service of Cosmo Bio Co., Ltd. (Tokyo, Japan). A synthetic peptide (C + EMQALNIINNIRQMQVVLER) derived from the FibA sequence of the *Spheniscus humboldti* predicted in this study was immunized a rabbit, and its serum was obtained. The serum was affinity-purified and used as the primary antibody. The antibodies against SAA and FibA were used at dilutions of 1:400 and 1:1,000, respectively. Antigen retrieval for SAA and FibA was performed by autoclaving in distilled water and pH 6.0 citrate buffer, respectively. The secondary antibody used was horseradish peroxidase-conjugated polymer anti-rabbit IgG antibody (Dako). Positive signals were visualized using 3,3’-diaminobenzidine tetrahydrochloride, according to the manufacturer’s protocol. Bovine AA amyloidosis was used as a positive control for the anti-SAA antibody. For negative controls, sections were incubated without the primary antibody. To assess nonspecific immunoreactivity of the anti-FibA antibody, normal tissues and plasma components within blood vessels were also evaluated. As a result, the nodular amyloid deposits observed in the liver, spleen, and heart were immunolabeled for FibA, while the diffuse amyloid deposits found in the perisinusoidal space of the liver, the vascular walls and red pulp of the spleen, the vascular walls and stroma of the kidneys and heart, the lamina propria of the esophagus and intestine, and the submucosa of the trachea were immunolabeled for SAA (Table 2; Fig. 1c and d, and Supplementary Figs. 2–5). No apparent co-localization of nodular and diffuse amyloid deposits was identified by comparison of serial sections. No FibA immunoreactivity was observed in the normal control tissues (e.g. hepatocytes) or plasma components within blood vessels (Supplementary Fig. 7). Based on these findings, the penguin was diagnosed with systemic amyloidosis involving concurrent deposition of FibA- and SAA-derived amyloids, as well as necrotizing granulomatous airsacculitis with intralesional hyphae. The cause of death was attributed to necrotizing granulomatous airsacculitis associated with suspected aspergillosis, although systemic amyloid deposition may also have contributed to the animal’s clinical deterioration.

**Table 2:** Distribution and precursor proteins of amyloid deposits in a Humboldt penguin

Organ	Severity of amyloid deposition *	
	Fibrinogen Aα-chain-derived amyloid	Amyloid A
Liver	++	+++
Spleen	+++	+++
Kidney	-	++
Heart	++	++
Lung **	-	-
Esophagus	-	++
Intestine	-	++
Trachea	-	+

*The presence and extent of amyloid deposition were assessed by Congo red staining, and amyloid typing in each tissue was based on immunohistochemistry. LC-MS/MS was used separately to confirm the amyloid precursor proteins

The severity of amyloid deposition is represented as follows: -, without deposition; +, mild deposition; ++, moderate deposition; +++, severe deposition

**Only a few small amyloid deposits were present in the lung and could not be typed

**Fig. 3:**
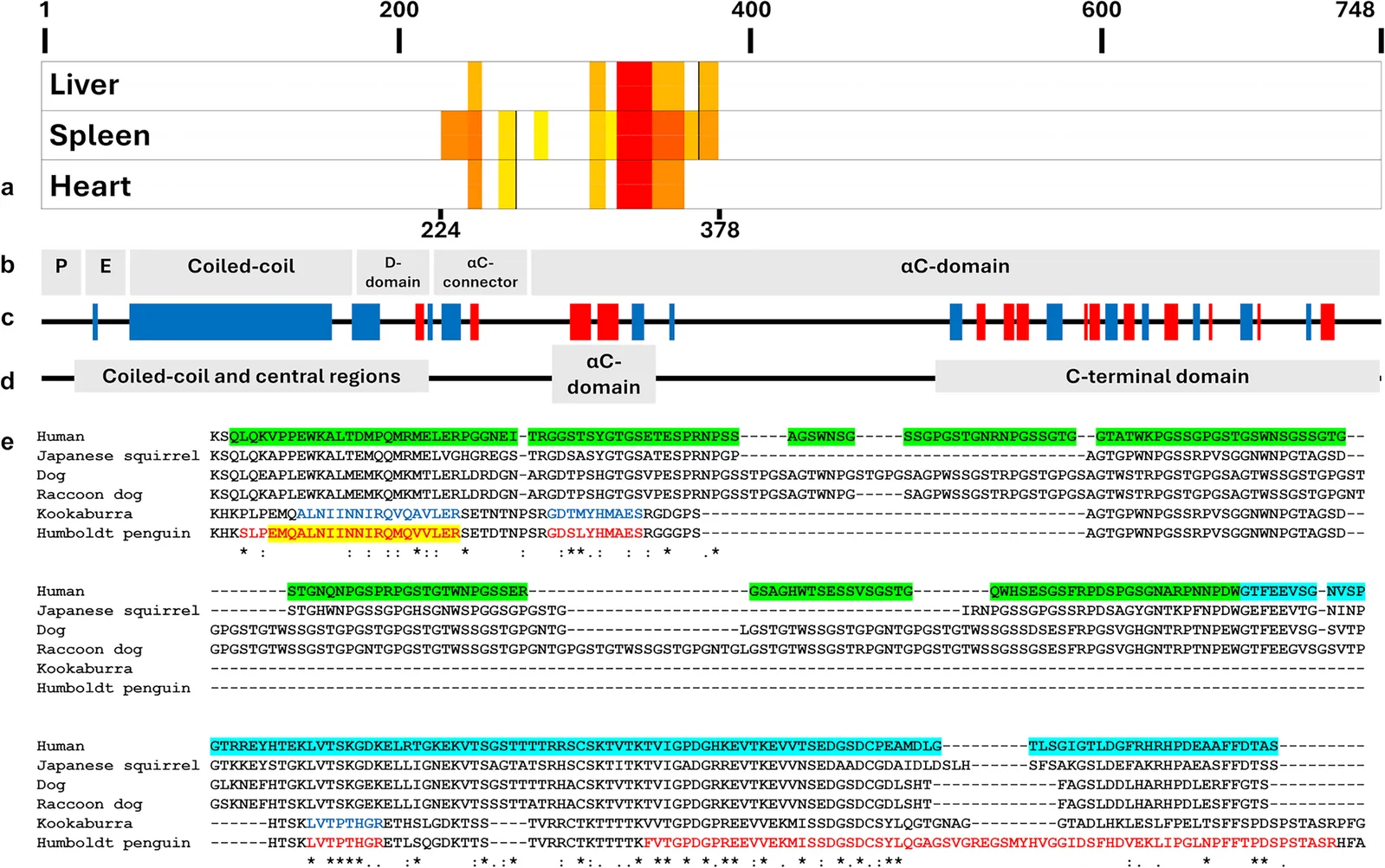
Amyloidogenic region in Humboldt penguin fibrinogen Aα-chain. (**a**) Peptides derived from FibA identified by mass spectrometry are mapped as color bars. Each bar is colored according to the total number of peptides detected in each organ, with warmer colors indicating a higher number of significantly detected peptides. White means no peptides were detected. Note that peptides spanning Ser224–Arg378 were frequently detected. The maximum numbers of detected peptides in each organ were 9, 12, and 7, respectively. The black vertical lines indicate non-tryptic truncation sites. (**b**) Predicted domain structure of Humboldt penguin FibA based on pairwise alignment with the human sequence. (**c**) Predicted secondary structure of Humboldt penguin FibA generated by AlphaFold3. Red bars indicate β-strands, and blue bars indicate α-helices. (**d**) Domain architecture of Humboldt penguin FibA annotated using InterPro. (**e**) Multiple sequence alignment of FibA from human, Japanese squirrel, dog, Japanese raccoon dog, blue-winged kookaburra, and Humboldt penguin. The sequences frequently detected in the Humboldt penguin amyloid (red letters) were located predominantly within the region corresponding to the human αC-connector (highlighted in green) to the αC-domain (highlighted in blue). The sequence highlighted in yellow corresponds to the immunogen used to generate the anti-FibA antibody employed in this study. Note that blue letters indicate sequences frequently detected in the kookaburra amyloid

On reanalyzing the LC-MS/MS data to identify the regions responsible for amyloid formation, peptides derived from the αC-region (Ser224–Arg378) of full-length FibA were detected at a high frequency in FibA-derived amyloid (Fig. 3a and b). Furthermore, structural prediction using AlphaFold3 (Abramson et al. 2024) indicated that some peptides in this region were predicted to form β-sheet structures (Fig. 3c). Domain prediction using InterPro (Blum et al. 2025) suggested that this region is part of the αC-domain (Fig. 3d). Multiple sequence alignment with mammalian FibA sequences revealed that the αC-connector of the penguin was shorter than those of mammals, and that the Humboldt penguin FibA peptides detected in the amyloid corresponded partly to the αC-connector but predominantly to the αC-domain (Fig. 3e). Similarly, pairwise alignment with human FibA showed that the detected peptides aligned partly with the αC-connector but predominantly with the αC-domain (Supplementary Fig. 8).

## Discussion and conclusion

In this study, mass spectrometry and IHC revealed the simultaneous deposition of FibA- and SAA-derived amyloids in a Humboldt penguin, leading to a diagnosis of double amyloidosis.

Previous studies in humans and animals have demonstrated FibA-derived amyloid deposition in multiple organs (Yazaki et al. 2018; Iwaide et al. 2023a; Murakami et al. 2026). In the present case, nodular deposits of FibA-derived amyloid were found in the perivascular regions of the liver, spleen, and heart. Although the liver, spleen, and heart are also common sites of AA deposition, the nodular morphology of FibA-derived amyloid observed in the present case was distinct from the diffuse pattern of AA deposition and may therefore provide a useful morphological clue for distinguishing the two types of amyloids; however, as this observation is based on a single case, further studies involving additional cases are required to determine whether this morphological distinction is consistently observed. Interestingly, some FibA amyloid deposits exhibited a spheroidal morphology and PAS positivity, features resembling those of corpora amylacea (Riba et al. 2021). However, these deposits were confirmed to be amyloid by Congo red staining and TEM and were associated with FibA by IHC and proteomic analysis. The PAS positivity suggests the presence of carbohydrate-containing components within or around these deposits, although their biochemical identity remains unknown. The absence of Alcian blue staining at both pH 1.0 and 2.5 suggests that acidic mucosubstances are not major components of these deposits.

From the N-terminus to the C-terminus, FibA consists of fibrinopeptide A, the E-domain, the coiled-coil region, the D-domain, and the αC-region, which comprises the αC-connector and αC-domain (Soria et al. 2019). The αC-region is known to constitute the amyloid fibrils in FibA-derived amyloidosis (Yazaki et al. 2018; Iwaide et al. 2025a). However, the predominant amyloid-forming region appears to differ among animal species. While the αC-domain primarily constitutes the amyloid in humans, Japanese squirrels and Japanese raccoon dogs, a region corresponding mainly to the αC-connector is dominantly detected in the amyloid in dog and blue-winged kookaburra (Yazaki et al. 2018; Iwaide et al. 2023a, 2025b; Murakami et al. 2026; Yoshikura et al. 2026). In the present case, the frequent detection of the αC-region (Ser224–Arg378) in FibA-derived amyloid suggests that a sequence spanning both the αC-connector and αC-domain, rather than either region alone, is a major constituent of the amyloid fibrils in penguin FibA amyloidosis (Fig. 3, Supplementary Tables 2 and 3). Notably, the corresponding sequence reported in the blue-winged kookaburra was restricted to Ala230–Arg283, which corresponds to αC-connector, indicating a difference in the C-terminal extent of the putative amyloid-forming region between these two avian species (Murakami et al. 2026). The αC-region shows considerable sequence variation across species, particularly within the αC-connector (Fig. 3). Therefore, sequence variation within the αC-region may contribute to species-specific differences in the amyloid-forming sequences of FibA.

Another methodological consideration in the present study is the incomplete annotation of the Humboldt penguin proteome. Because the initially available predicted FibA sequence was incomplete, we independently reconstructed the FibA sequence from the Humboldt penguin genome using the orthologous sequence of the closely related emperor penguin as a reference. However, protein sequences reconstructed using such a reference-based approach remain predicted sequences; therefore, validation by an independent method, such as PCR followed by sequencing, would ideally be required. Notably, the sequence reconstructed in the present study was 100% identical to the Humboldt penguin FibA sequence subsequently deposited in a public database (NCBI accession: KAM6266820), thereby supporting the validity of our sequence prediction. These findings highlight both the potential limitations of automated protein annotation in non-model species and the utility of reference-guided reannotation for the proteomic identification of amyloid precursor proteins.

FibA-derived amyloid has been reported to predominantly deposit in the renal glomeruli and small cardiac vessels in humans, Japanese squirrels, dogs, Japanese raccoon dogs, and a blue-winged kookaburra (Yazaki et al. 2018; Iwaide et al. 2023a; Kobayashi et al. 2026; Murakami et al. 2026; Yoshikura et al. 2026). In contrast, no FibA-derived amyloid deposition was observed in the kidney in the present case. Furthermore, the FibA-derived amyloid deposition in the spleen and liver in the present case is not typical of those reported in human and animal cases, suggesting that the predominant sites of amyloid deposition may differ among species even when the amyloid is derived from the same αC-region of FibA. One possible explanation for this difference is that the sequences within the αC-region constituting the amyloid fibrils vary among species, and differences in the amyloidogenic regions may influence the tissue distribution of FibA-derived amyloid. Further accumulation of cases will be necessary to clarify the distribution of FibA-derived amyloid deposition and the mechanisms underlying its tissue tropism in penguins.

AA amyloidosis is a systemic amyloidosis caused by deposition of AA and is the most common form of amyloidosis in animals (Iwaide et al. 2025a). Elevated levels of serum SAA associated with chronic inflammation are considered a key factor in the onset of AA amyloidosis (Murakami et al. 2014; Westermark et al. 2015). In this case, necrotizing granulomatous airsacculitis with intralesional fungal hyphae was present, suggesting that *Aspergillus* infection may have been associated with the elevated serum SAA levels and AA deposition. AA amyloidosis frequently occurs in aquatic birds, including mute swans and flamingos, often in association with chronic inflammatory conditions (Tanaka et al. 2008; Ono et al. 2020). In penguins, systemic amyloidosis has been reported in an African penguin; however, the amyloid precursor proteins were not determined (Kim et al. 2022). To clarify the epidemiological details of amyloidosis in penguins, it is important to accumulate further case reports.

In systemic amyloidosis, plasma-derived proteins such as FibA and SAA form amyloid, and their deposition can extend to multiple organs (Buxbaum et al. 2024). In animals, AA amyloidosis associated with chronic inflammation has traditionally been considered the most common form of systemic amyloidosis (Woldemeskel 2012; Iwaide et al. 2025a). On the other hand, since fibrinogen is a positive acute-phase protein whose plasma concentration rises in association with inflammation (Valdivia et al. 2020), it has been suggested that an increase in circulating FibA concentrations related to inflammation may constitute a predisposing factor for FibA amyloidosis (Murakami et al. 2026). To clarify the exact risk of developing FibA and AA amyloidosis, it is important to accumulate more cases in the future and identify detailed predisposing factors. Furthermore, since genetic mutations are known to be risk factors for FibA amyloidosis in humans, the role of mutations in penguins should also be elucidated in the future (Benson et al. 1993; Yazaki et al. 2018).

In conclusion, we diagnosed the first reported case of FibA amyloidosis in a Humboldt penguin and, to our knowledge, the first reported case of systemic double amyloidosis involving two amyloid subtypes in a bird.

## Supplementary Information

Below is the link to the electronic supplementary material.

Supplementary Material 1

## Data Availability

All data supporting the findings of this study are available within the paper and its Supplementary Information.
