# Supplementary material for: Concurrent fibrinogen Aα-chain amyloidosis and AA amyloidosis in a Humboldt penguin (Spheniscus humboldti)
Source: Vet Res Commun. 2026 Oct 10;50(6):626. doi: 10.1007/s11259-026-11572-y (PMC13653883; doi:10.1007/s11259-026-11572-y)
Supplement: Supplementary file 1 [file 11259_2026_11572_MOESM1_ESM.pdf]

Supplementary Information for

Concurrent fibrinogen Aα-chain amyloidosis and AA amyloidosis in a Humboldt penguin (*Spheniscus humboldti*)

Susumu Iwaide[1,2,3]*, Natsumi Kobayashi[2], Mako Ikudome[2], Shiho Takezawa[4], Yoshiyuki Itoh[5], Tomoyuki Shibahara[3,6], Keisuke Kawase[7], Tomoaki Murakami[2]

[1]Research Unit of Innovative One Medicine, Advanced Research Center for One Welfare, Tokyo University of Agriculture and Technology, 3-5-8 Saiwai-cho, Fuchu, Tokyo, Japan; [2]Laboratory of Veterinary Toxicology, Tokyo University of Agriculture and Technology, 3-5-8 Saiwai-cho, Fuchu, Tokyo, Japan; [3]National Institute of Animal Health, National Agriculture and Food Research Organization, 3-1-5 Kannondai, Tsukuba, Ibaraki, Japan; [4]Ibaraki Prefectural Kenpoku Livestock Hygiene Service Center, 966-1 Nakagachi-cho, Mito, Ibaraki, Japan; [5]Smart-Core-Facility Promotion Organization, Tokyo University of Agriculture and Technology, 3-5-8 Saiwai-cho, Fuchu, Tokyo, Japan; [6]Department of Veterinary Immunology, Graduate School of Veterinary Medical Sciences, Osaka Metropolitan University, 1-58 Rinku-oraikita, Izumisano, Osaka, Japan; [7]Hitachi Kamine Zoo, 5-2-22, Miyata-cho, Hitachi, Ibaraki, Japan

***Correspondence:** Susumu Iwaide, Research Unit of Innovative One Medicine, Advanced Research Center for One Welfare, Tokyo University of Agriculture and Technology, 3-5-8 Saiwai-cho, Fuchu, Tokyo, Japan;
Tel: +81-42-367-5883; E-mail: fq3733@go.tuat.ac.jp.

This file includes the following:
Supplementary Figs. 1–8
Supplementary Tables 1–3

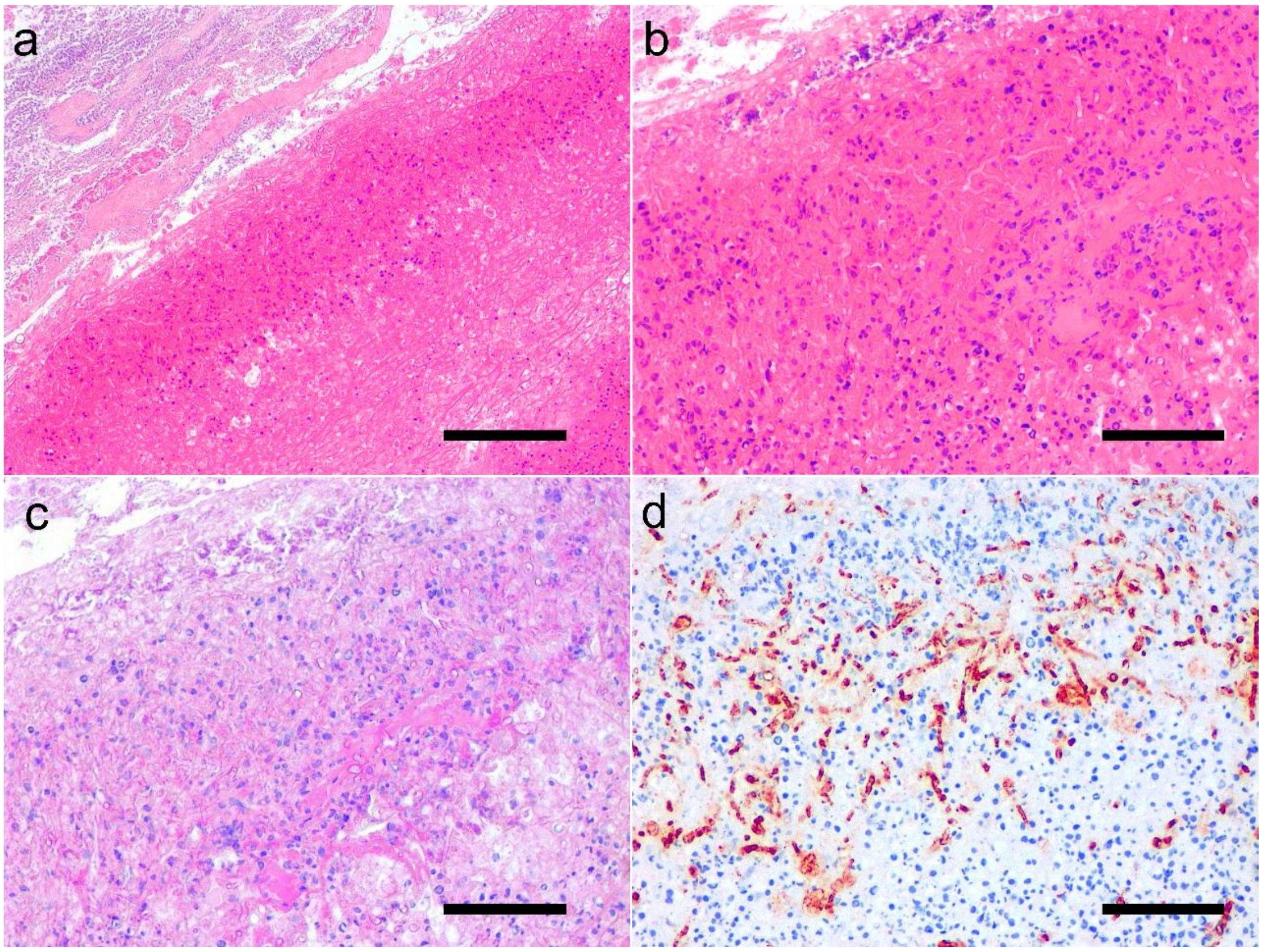

Supplementary Fig. 1. Air sac, Humboldt penguin. Necrosis and debris are present in the mucosa (a, b). Hyphae that stained positive for the periodic acid-Schiff reaction are present in the area (b, c). The hyphae are immunolabeled for anti-*Aspergillus* spp. antibody. Immunohistochemistry for *Aspergillus* spp. Bars = 100 µm (a), 50 µm (b–d).

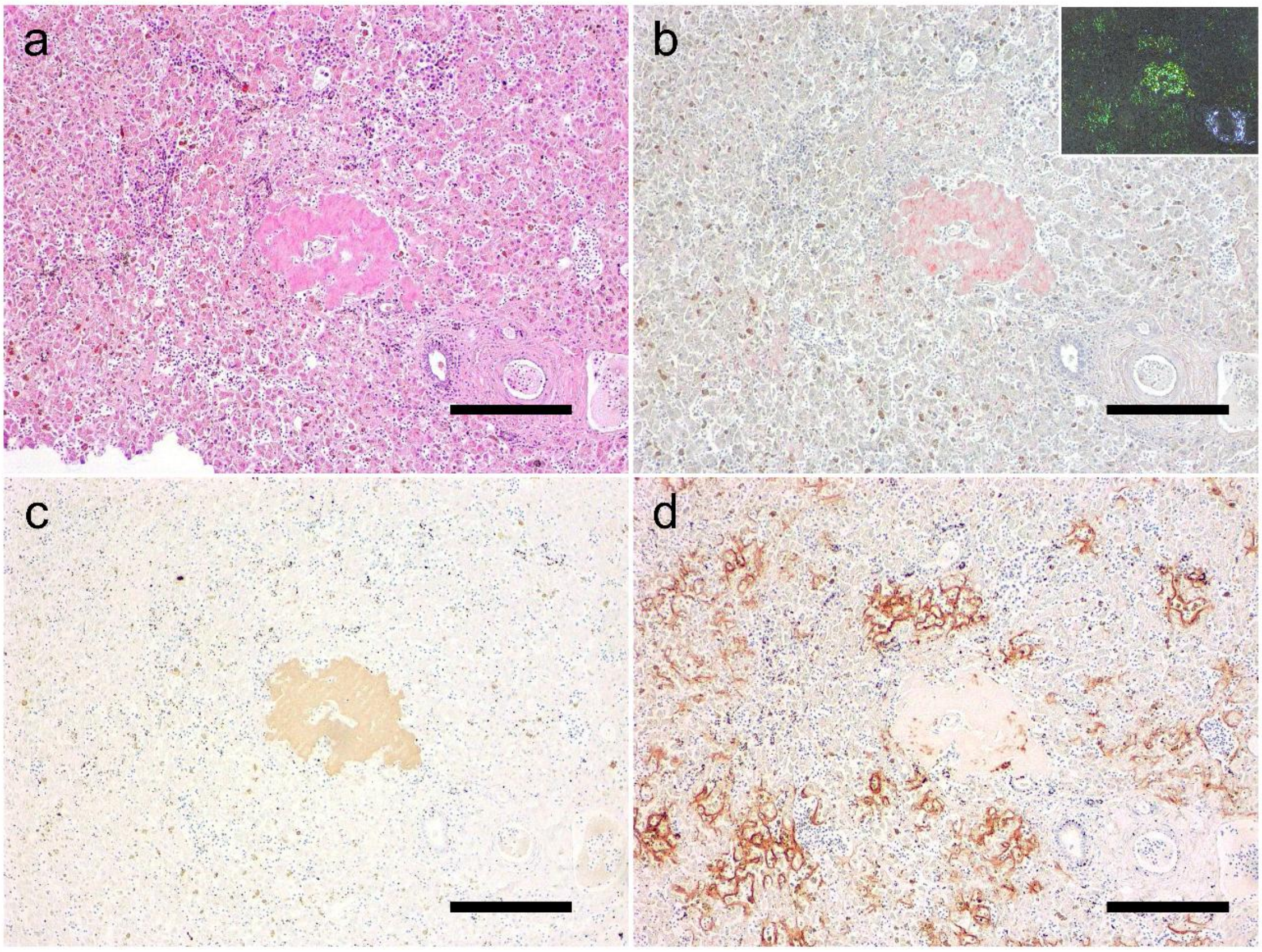

Supplementary Fig. 2. Liver, Humboldt penguin. Nodular amyloid deposits are present in the liver. In addition, diffuse amyloid deposits are present in the perisinusoidal spaces (a). Both types of amyloid deposits stain pink-red (b) in Congo red and show yellow-green birefringence under polarized light (b, inset). Nodular and diffuse amyloid deposits are specifically positive with anti-fibrinogen Aα-chain (FibA) antibody (c) and anti-serum amyloid A (SAA) antibody (d), respectively. Immunohistochemistry for FibA (c) and SAA (d). Bars = 200 μm.

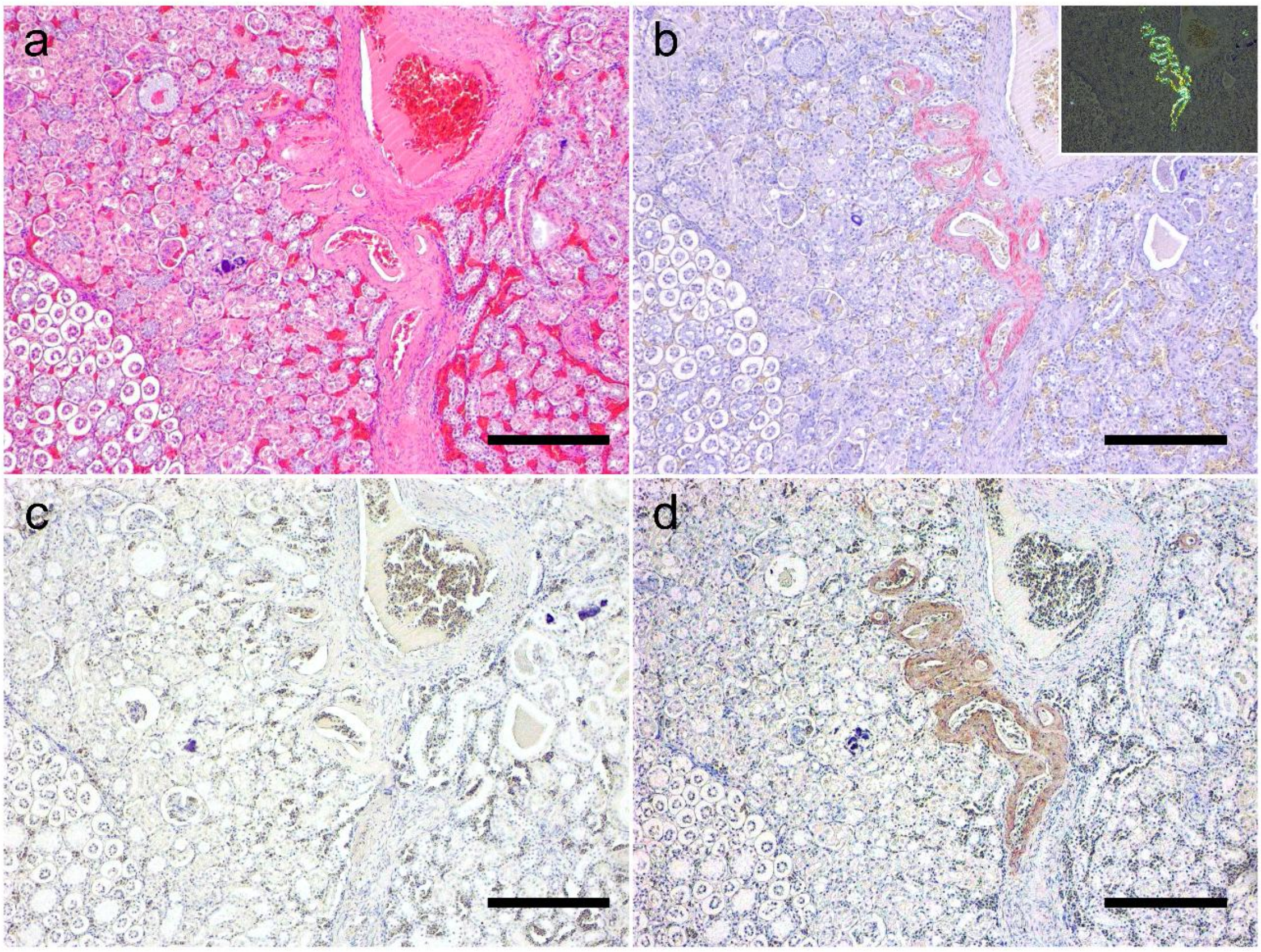

Supplementary Fig. 3. Kidney, Humboldt penguin. Diffuse amyloid deposits are present in the vascular wall (a). These amyloids stain pink-red (b) in Congo red and show yellow-green birefringence under polarized light (b, inset). Amyloid deposits are negative with anti-FibA antibody (c) but positive with anti-SAA antibody (d). Immunohistochemistry for FibA (c) and SAA (d). Bars = 200 µm.

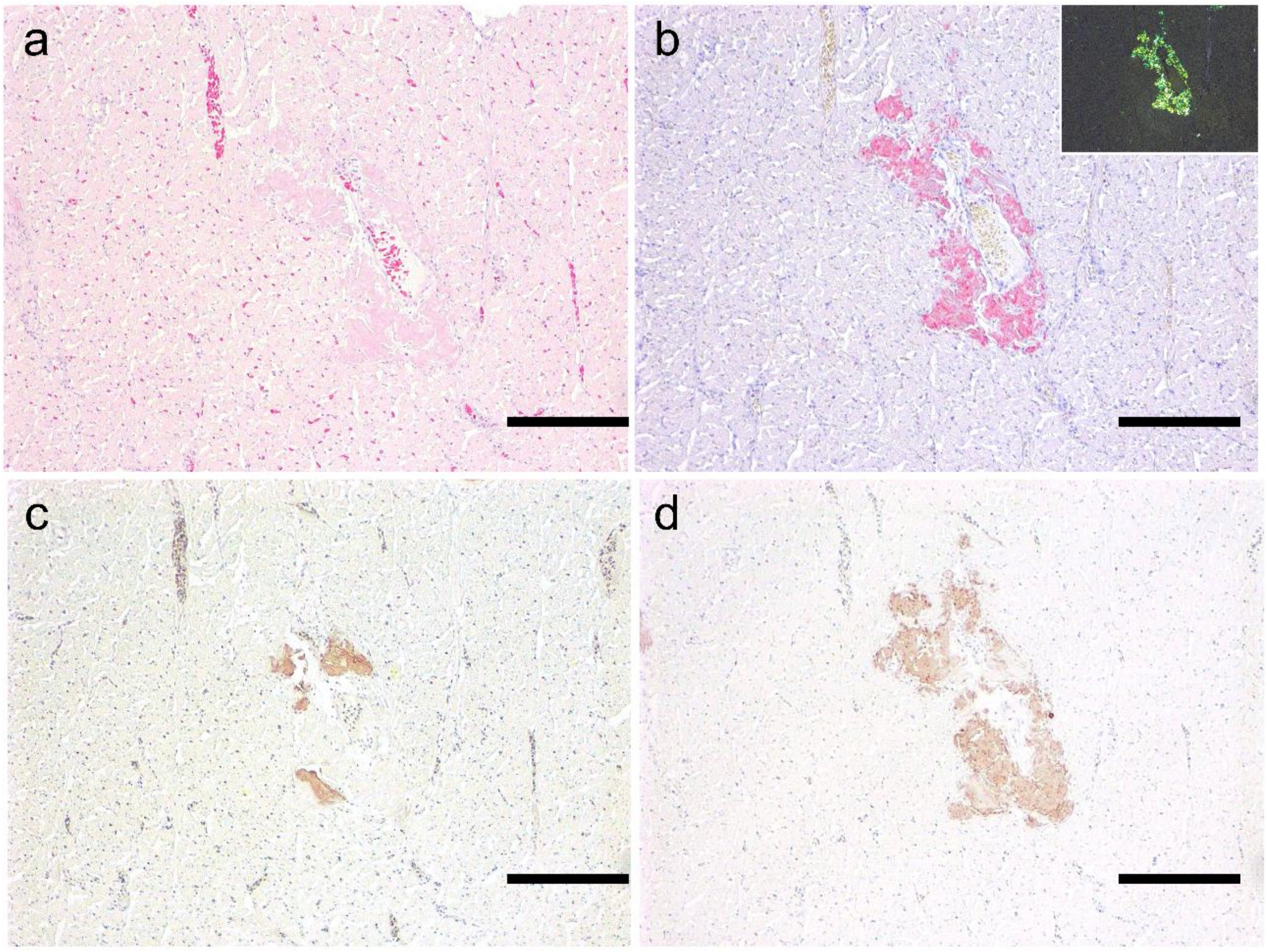

Supplementary Fig. 4. Heart, Humboldt penguin. Nodular amyloid deposits are present in the heart (a). In addition, diffuse amyloid deposits are present in the myocardial stroma and around vessels (a). Both types of amyloid deposits stain pink-red (b) in Congo red and show yellow-green birefringence under polarized light (b, inset). Nodular and diffuse amyloid deposits are specifically positive with anti-FibA antibody (c) and anti-SAA antibody (d), respectively. Immunohistochemistry for FibA (c) and SAA (d). Bars = 200 μm.

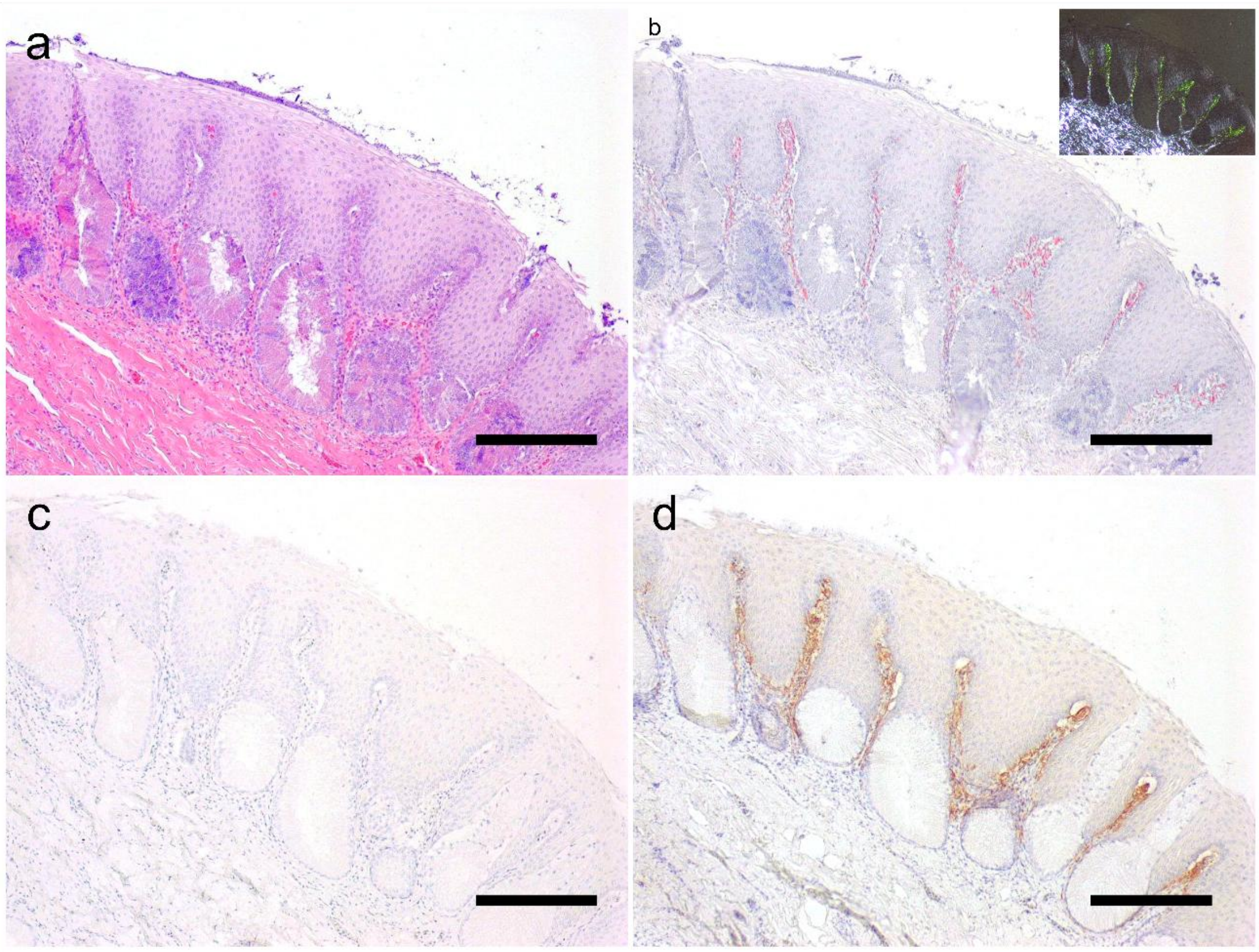

Supplementary Fig. 5. Esophagus, Humboldt penguin. Diffuse amyloid deposits are present in the lamina propria (a). These amyloids stain pink-red (b) in Congo red and show yellow-green birefringence under polarized light (b, inset). Amyloid deposits are not negative with anti-FibA antibody but positive with anti-SAA antibody (d). Immunohistochemistry for FibA (c) and SAA (d). Bars = 200 μm.

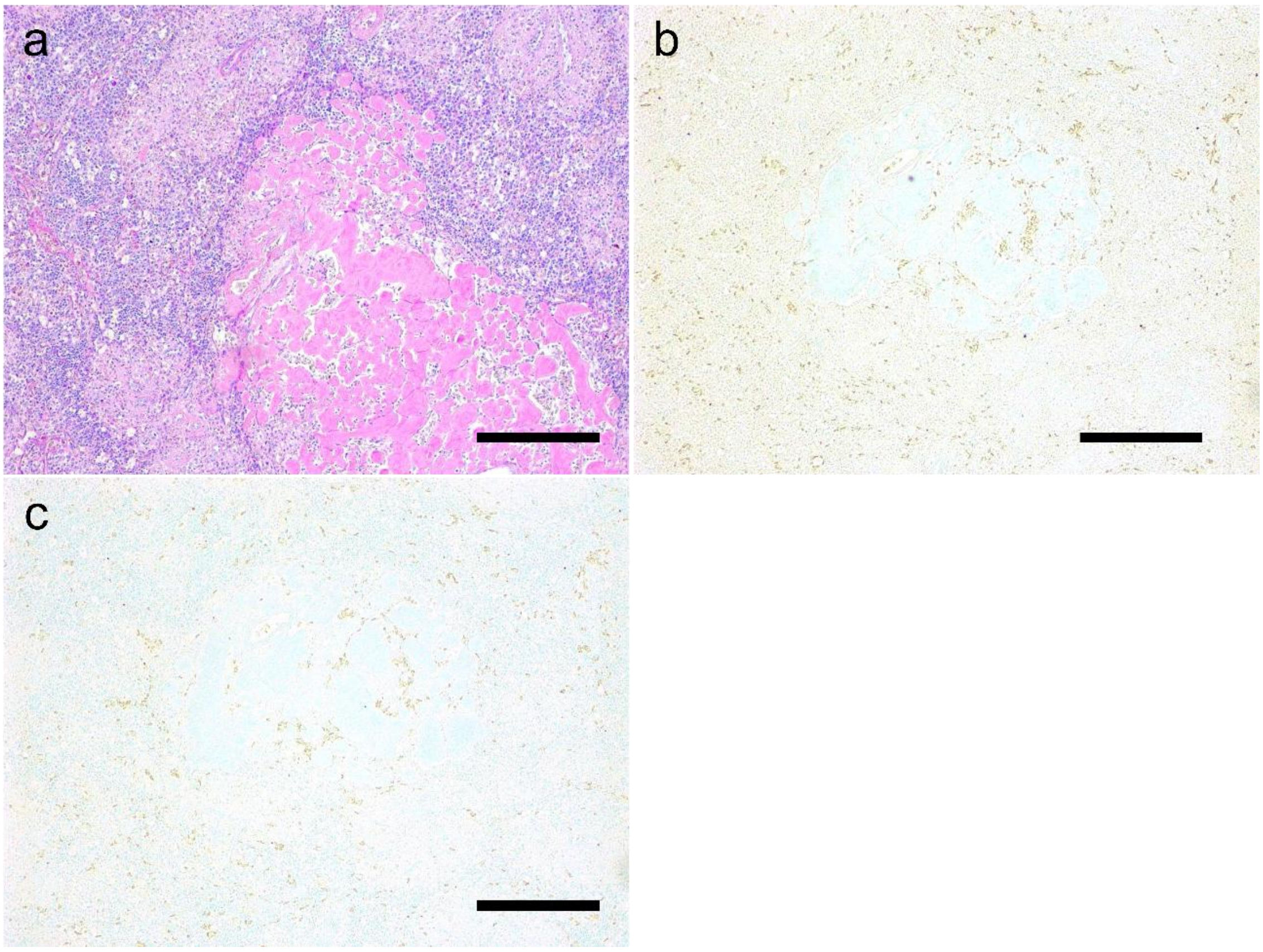

Supplementary Fig. 6. Spleen, Humboldt penguin. Nodular amyloid deposits are positive with periodic acid-Shiff stain (a), but are negative for Alcian blue at both pH 1.0 (b) and 2.5 (c). Bars = 200 µm.

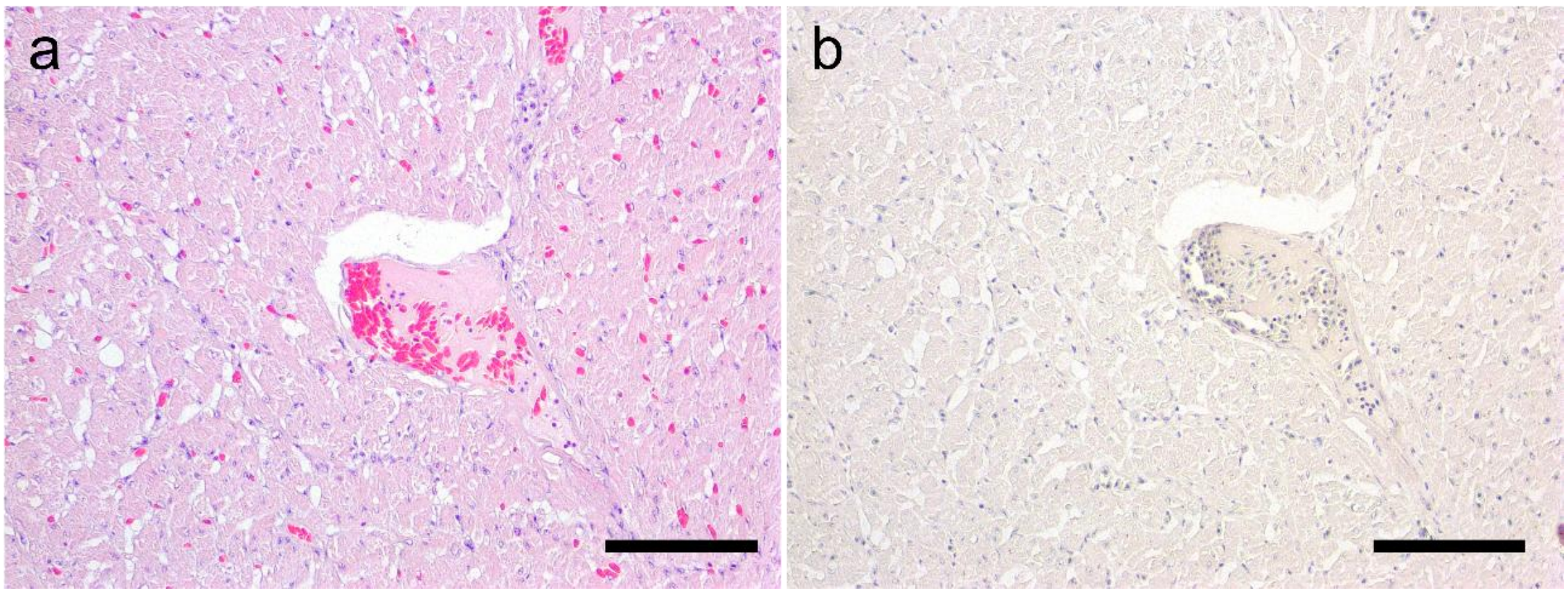

Supplementary Fig. 7. Heart, Humboldt penguin. No immunoreactivity for FibA was observed in the plasma within blood vessels (b). Hematoxylin and eosin (a), and immunohistochemistry for FibA (b). Bars = 200 μm.

```
Human          1 ADSGEGDFLAEGGGVRGPRVVER-HQSACK-DSDWPFCSDEDWNYKCPSG   48
                  ..||..|..||.|||||||:||. .||.|: :.:||.|:|:||..|||||
Penguin        1 -QDGESTFEKEGAGVRGPRIVEHMSQSTCQYEKNWPICADDDWGTKCPSG   49

Human         49 CRMKGLIDEVNQDFTNRINKLKNSLFEYQKNNKDSHSLTTNIMEILRGDF   98
                 |||:|||||.:||:::||:|:|..|.|.|.|.|.|.|:.:....:.:|:.:.
Penguin       50 CRMQGLIDETDQDYSHRIDKIKKLLTENQNNYKKSNRIIVETINVLKPNL   99

Human         99 SSANNRDNTYNRVSEDLRSRIEVLKRKVIEKVQHIQLLQKNVRAQLVDMK  148
                 .||...|.||:.||.:||.||..||::|.:|..|:.||.:::.|:|:||
Penguin      100 DSAQQIDETYDHVSGELRRRIVTLKQRVVTQVNRIKALQSSIQEQVVEMK  149

Human        149 RLEVDIDIKIRSCRGSCSRALAREVDLKDYEDQQKQLEQVIAKDLLPSRD  198
                 ||.||||||||:|:|:|:|:...:||.:.|::.||||.|..:.:|.|...
Penguin      150 RLXVDIDIKIRACKGTCARSFDYQVDKESYDNIQKQLTQANSINLHPELQ  199

Human        199 RQHLPLIKMKPVPDL-VPGNFKSQLQKVPPEWKAL---TDMPQMRMELER  244
                 ...:..:|||.:.|. ||.:||   .|..||.:||   .::.||::.|||
Penguin      200 TITMSTLKMKQLKDSNVPEHFK---HKSLPEMQALNIINNIRQMQVVLER  246

Human        245 PGGNEITRGGSTSYGTGSETESPRNPSSAGSWNSGSSGPGSTGNRNPGSS  294
                                  |||::  |||
Penguin      247 -----------------SETDT--NPS-----------------------  254

Human        295 GTGGTATWKPGSSGPGSTGSWNSGSSGTGSTGNQNPGSPRPGSTGTWNPG  344

Penguin      255 --------------------------------------------------  254

Human        345 SSERGSAGHWTSESSVSGSTGQWHSESGSFRPDSPGSGNARPNNPDWGTF  394
                    ||.:.:.:.:||...|.:
Penguin      255 ---RGDSLYHMAESRGGGPS------------------------------  271

Human        395 EEVSGNVSPGTRREYHTEKLVTSKGDKELRTGKEKVTSG-STTTTRRSCS  443
                                ||.||||.      ..|:|.::.| .||:|.|.|:
Penguin      272 ---------------HTSKLVTP------THGRETLSQGDKTTSTVRRCT  300

Human        444 K-TVTKTVIGPDGHKEVTKEVVTSEDGSDCPEAMDLGTLS------GIGT  486
                 | |.||.|.||||.:|...|.:.|.|||||......|::.      .:|.
Penguin      301 KTTTTKFVTGPDGPREEVVEKMISSDGSDCSYLQGAGSVGREGSMYHVGG  350

Human        487 LDGFRHRH---PDEAAFF--DTAST-GKTFPGFFSPMLGEFVSETESRGS  530
                 :|.|....    |....||  |:.|| .:.|.|..|......|:.|.|...
Penguin      351 IDSFHDVEKLIPGLNPFFTPDSPSTASRHFAGSSSISTSSHVTGTGSSHL  400

Human        531 ESGI------------FTNTKESSSH-----HPGIAEFPSRGKSSSYSKQ  563
                 .:|:            ||:..|....      |...:.||||  ||:|:||.
Penguin      401 STGVSSHSGAYGGKDKFTDLGEEEEDDFGGLHLQPSGFPSR--SASHSKT  448

Human        564 FTSST--SYNRGDSTFESKSYK---MADEAGSEADHEGTHSTKRGHAKSR  608
                 ..:||  |:|:|.||||:||.|    :|::.|.....:....|....|:|.
Penguin      449 VVTSTSSSFNKGGSTFETKSLKTREIAEQLGGVQHDQSAEDTPDFQARSS  498

Human        609 PVRGI--HTSPLGKPSLSP-------------------------------  625
                 ...|:  .|:..||.....
Penguin      499 RPSGVKQRTANTGKDCDDIRQKHTFGVKSGVFKIKPAGSNKVLSVYCDQE  548

Human        626 --------------------------------------------------  625

Penguin      549 TTLGGWLLIQQRMDGSVNFNRTWQDYKRGFGSVDGRGRGEFWLGNENIHL  598

Human        626 --------------------------------------------------  625

Penguin      599 LTQNDTLLRVELEDWDGNAVYAEYIIQVGSEAEGYALAVSSYEGTAGDAL  648

Human        626 --------------------------------------------------  625

Penguin      649 IAGWLEEGTEYTSHAQMQFSTFDRDQDRWEESCAEMYGGGWWYNSCQAAN  698

Human        626 --------------------------------------------------  625

Penguin      699 LNGIYYLGGHYDPRYNIPYEIENGVVWLPFKASDYSLKIVRMKIRPIETL  748
```

Supplementary Fig. 8. Pairwise alignment of FibA from human and Humboldt penguin. The structural regions of the Humboldt penguin FibA sequence were assigned based on homology with the human FibA sequence. The regions are color-coded as follows: fibrinopeptide A (purple), E-domain (gray), coiled-coil region (yellow), D-domain (green), αC-connector (pink), and αC-domain (light blue). Peptides identified by mass spectrometry are shown in red within the penguin FibA sequence. Black arrows indicate the starting point of the amyloid-forming region in human FibA. Black-colored residues in the human sequence represent mutation sites associated with human FibA amyloidosis. These mutations are clustered within residues 517–555 of the αC-domain, which is considered the amyloidogenic segment of human FibA.

Supplementary Table 1. Proteins detected by LC-MS/MS analysis of amyloid deposits using a protein database derived from the genome assembly (GCA_027474245).

Amyloid morphology	Organ	Accession	Protein name	Score	Sequences	emPAI*
Nodular amyloid	Liver	KAF1426833.1	Vitronectin, partial	310	19	0.55
Nodular amyloid	Liver	KAF1410912.1	Hemoglobin subunit beta, partial	162	5	0.75
Nodular amyloid	Liver	KAF1426285.1	Chromodomain-helicase-DNA-binding protein 3, partial	123	12	0.02
Nodular amyloid	Liver	KAF1422510.1	Trypsin, partial	107	2	0.38
Nodular amyloid	Liver	KAF1404247.1	Keratin, type I cytoskeletal 19, partial	98	4	0.27
Nodular amyloid	Liver	KAF1409665.1	Keratin, type II cytoskeletal 6A, partial	88	2	0.1
Nodular amyloid	Liver	KAF1409671.1	Keratin, type II cytoskeletal 75, partial	65	3	0.05
Nodular amyloid	Liver	KAF1398448.1	Asporin, partial	50	5	0.08
Nodular amyloid	Liver	KAF1405730.1	Fibrinogen alpha chain, partial	47	2	0.11
Nodular amyloid	Liver	KAF1405847.1	Bactericidal permeability-increasing protein, partial	41	1	0.07
Nodular amyloid	Liver	KAF1402331.1	Histone H2A type 2-B, partial	41	1	0.25
Nodular amyloid	Liver	KAF1428000.1	Plasminogen, partial	34	1	0.04
Nodular amyloid	Liver	KAF1417154.1	Hemoglobin subunit alpha, partial	30	3	0.48
Nodular amyloid	Liver	KAF1409669.1	Keratin, type II cytoskeletal 75, partial	30	1	0.06
Nodular amyloid	Liver	KAF1407927.1	GTP-binding protein 2, partial	29	1	0.05
Nodular amyloid	Liver	KAF1403199.1	Histone H2B 5, partial	29	2	0.24
Nodular amyloid	Liver	KAF1406489.1	Histidine--tRNA ligase, cytoplasmic, partial	28	1	0.07
Nodular amyloid	Liver	KAF1405169.1	Transcription initiation factor TFIID subunit 4, partial	26	1	0.11
Nodular amyloid	Liver	KAF1429209.1	Apolipoprotein A-I, partial	19	1	0.11
Nodular amyloid	Liver	KAF1418258.1	Phospholipid-transporting ATPase IB, partial	18	1	0.02
Nodular amyloid	spleen	KAF1426833.1	Vitronectin, partial	882	42	0.99
Nodular amyloid	spleen	KAF1405730.1	Fibrinogen alpha chain, partial	269	15	0.5
Nodular amyloid	spleen	KAF1426285.1	Chromodomain-helicase-DNA-binding protein 3, partial	106	10	0.02
Nodular amyloid	spleen	KAF1429209.1	Apolipoprotein A-I, partial	87	1	0.11
Nodular amyloid	spleen	KAF1405847.1	Bactericidal permeability-increasing protein, partial	81	3	0.07
Nodular amyloid	spleen	KAF1429220.1	Apolipoprotein A-IV, partial	78	4	0.27
Nodular amyloid	spleen	KAF1410900.1	Hemoglobin subunit epsilon, partial	62	4	0.45

Nodular amyloid	spleen	KAF1427924.1	Actin, alpha skeletal muscle, partial	38	2	0.08
Nodular amyloid	spleen	KAF1417154.1	Hemoglobin subunit alpha, partial	29	1	0.22
Nodular amyloid	spleen	KAF1419913.1	Exportin-2, partial	21	1	0.03
Nodular amyloid	spleen	KAF1428901.1	Receptor-type tyrosine-protein phosphatase U, partial	18	1	0.02
Nodular amyloid	spleen	KAF1415968.1	Leucine--tRNA ligase, cytoplasmic, partial	16	1	0.32
Nodular amyloid	Heart	KAF1426833.1	KAF1426833.1 Vitronectin, partial	175	10	0.28
Nodular amyloid	Heart	KAF1419813.1	KAF1419813.1 Actin, alpha skeletal muscle B, partial	158	9	0.46
Nodular amyloid	Heart	KAF1403990.1	KAF1403990.1 Actin, gamma-enteric smooth muscle, partial	133	10	0.35
Nodular amyloid	Heart	KAF1426285.1	KAF1426285.1 Chromodomain-helicase-DNA-binding protein 3, partial	101	13	0.02
Nodular amyloid	Heart	KAF1415886.1	KAF1415886.1 Myoglobin, partial	81	4	0.69
Nodular amyloid	Heart	KAF1405730.1	KAF1405730.1 Fibrinogen alpha chain, partial	77	4	0.11
Nodular amyloid	Heart	KAF1428000.1	KAF1428000.1 Plasminogen, partial	49	2	0.04
Nodular amyloid	Heart	KAF1412642.1	KAF1412642.1 Kallikrein-14, partial	45	1	0.4
Nodular amyloid	Heart	KAF1430687.1	KAF1430687.1 Collagen alpha-1(II) chain, partial	41	2	0.03
Nodular amyloid	Heart	KAF1398448.1	KAF1398448.1 Asporin, partial	38	1	0.08
Nodular amyloid	Heart	KAF1397937.1	KAF1397937.1 Sn1-specific diacylglycerol lipase beta, partial	35	1	0.04
Nodular amyloid	Heart	KAF1402331.1	KAF1402331.1 Histone H2A type 2-B, partial	34	1	0.25
Nodular amyloid	Heart	KAF1403199.1	KAF1403199.1 Histone H2B 5, partial	29	1	0.24
Nodular amyloid	Heart	KAF1396498.1	KAF1396498.1 Forkhead box protein O3, partial	27	2	0.06
Nodular amyloid	Heart	KAF1414311.1	KAF1414311.1 Histone H4, partial	26	1	0.3
Nodular amyloid	Heart	KAF1405169.1	KAF1405169.1 Transcription initiation factor TFIID subunit 4, partial	23	1	0.11
Nodular amyloid	Heart	KAF1421293.1	KAF1421293.1 DNA (cytosine-5)-methyltransferase 1, partial	20	1	0.21
Nodular amyloid	Heart	KAF1423209.1	KAF1423209.1 LIM domain-binding protein 1, partial	18	1	0.07
Nodular amyloid	Heart	KAF1408707.1	KAF1408707.1 Mucin-5AC, partial	13	1	0.03
Diffuse amyloid	Liver	KAF1397894.1	Actin, cytoplasmic 1, partial	220	15	0.7
Diffuse amyloid	Liver	KAF1422510.1	Trypsin, partial	153	4	0.38
Diffuse amyloid	Liver	KAF1426285.1	Chromodomain-helicase-DNA-binding protein 3, partial	110	14	0.02
Diffuse amyloid	Liver	KAF1402331.1	Histone H2A type 2-B, partial	98	4	0.56
Diffuse amyloid	Liver	KAF1429209.1	Apolipoprotein A-I, partial	82	3	0.38
Diffuse amyloid	Liver	KAF1414311.1	Histone H4, partial	81	4	0.7
Diffuse amyloid	Liver	KAF1419114.1	Annexin A5, partial	57	1	0.09
Diffuse amyloid	Liver	KAF1408665.1	Serum amyloid A protein, partial	55	2	0.24

Diffuse amyloid	Liver	KAF1410885.1	Hemoglobin subunit beta, partial	53	2	0.21
Diffuse amyloid	Liver	KAF1401624.1	histone H3.3, partial	48	2	0.22
Diffuse amyloid	Liver	KAF1398448.1	Asporin, partial	45	4	0.08
Diffuse amyloid	Liver	KAF1428000.1	Plasminogen, partial	40	1	0.04
Diffuse amyloid	Liver	KAF1403199.1	Histone H2B 5, partial	36	3	0.24
Diffuse amyloid	Liver	KAF1407490.1	Peptidyl-prolyl cis-trans isomerase A, partial	22	1	0.22
Diffuse amyloid	Liver	KAF1421293.1	DNA (cytosine-5)-methyltransferase 1, partial	20	1	0.21
Diffuse amyloid	Spleen	KAF1422510.1	Trypsin, partial	154	6	0.62
Diffuse amyloid	Spleen	KAF1426285.1	Chromodomain-helicase-DNA-binding protein 3, partial	122	13	0.02
Diffuse amyloid	Spleen	KAF1409665.1	Keratin, type II cytoskeletal 6A, partial	91	2	0.1
Diffuse amyloid	Spleen	KAF1408665.1	Serum amyloid A protein, partial	73	2	0.54
Diffuse amyloid	Spleen	KAF1404247.1	Keratin, type I cytoskeletal 19, partial	72	2	0.17
Diffuse amyloid	Spleen	KAF1428000.1	Plasminogen, partial	58	2	0.04
Diffuse amyloid	Spleen	KAF1409671.1	Keratin, type II cytoskeletal 75, partial	56	2	0.05
Diffuse amyloid	Spleen	KAF1427924.1	Actin, alpha skeletal muscle, partial	41	2	0.08
Diffuse amyloid	Spleen	KAF1402331.1	Histone H2A type 2-B, partial	39	1	0.25
Diffuse amyloid	Spleen	KAF1398448.1	Asporin, partial	37	3	0.08
Diffuse amyloid	Spleen	KAF1410885.1	Hemoglobin subunit beta, partial	34	1	0.21
Diffuse amyloid	Spleen	KAF1397809.1	Neuronal pentraxin-2, partial	32	1	0.06
Diffuse amyloid	Spleen	KAF1406489.1	Histidine--tRNA ligase, cytoplasmic, partial	27	2	0.07
Diffuse amyloid	Spleen	KAF1403199.1	Histone H2B 5, partial	26	1	0.24
Diffuse amyloid	Spleen	KAF1428901.1	Receptor-type tyrosine-protein phosphatase U, partial	23	1	0.02
Diffuse amyloid	Spleen	KAF1405169.1	Transcription initiation factor TFIID subunit 4, partial	23	1	0.11
Diffuse amyloid	Spleen	KAF1396583.1	Activating signal cointegrator 1 complex subunit 3, partial	19	1	0.01
Diffuse amyloid	Spleen	KAF1426833.1	Vitronectin, partial	17	1	0.06
Diffuse amyloid	Spleen	KAF1399997.1	NADPH oxidase 1, partial	14	2	0.05
Diffuse amyloid	Heart	KAF1408665.1	Serum amyloid A protein, partial	363	13	1.37
Diffuse amyloid	Heart	KAF1427924.1	Actin, alpha skeletal muscle, partial	314	16	0.57
Diffuse amyloid	Heart	KAF1419813.1	Actin, alpha skeletal muscle B, partial	274	14	0.57
Diffuse amyloid	Heart	KAF1409285.1	Myosin-6, partial	281	8	0.09
Diffuse amyloid	Heart	KAF1426833.1	Vitronectin, partial	171	9	0.37
Diffuse amyloid	Heart	KAF1415886.1	Myoglobin, partial	132	9	1.01

Diffuse amyloid	Heart	KAF1422510.1	Trypsin, partial	129	4	0.38
Diffuse amyloid	Heart	KAF1426285.1	Chromodomain-helicase-DNA-binding protein 3, partial	101	9	0.02
Diffuse amyloid	Heart	KAF1412736.1	Tropomyosin beta chain, partial	62	2	0.1
Diffuse amyloid	Heart	KAF1410900.1	Hemoglobin subunit epsilon, partial	44	2	0.21
Diffuse amyloid	Heart	KAF1398448.1	Asporin, partial	41	3	0.08
Diffuse amyloid	Heart	KAF1405625.1	Desmin, partial	40	2	0.14
Diffuse amyloid	Heart	KAF1419812.1	Cysteine and glycine-rich protein 3, partial	39	1	0.16
Diffuse amyloid	Heart	KAF1428000.1	Plasminogen, partial	37	1	0.04
Diffuse amyloid	Heart	KAF1415968.1	Leucine--tRNA ligase, cytoplasmic, partial	32	2	0.32
Diffuse amyloid	Heart	KAF1428045.1	Mucin-5B, partial	32	1	0.01
Diffuse amyloid	Heart	KAF1417154.1	Hemoglobin subunit alpha, partial	31	2	0.22
Diffuse amyloid	Heart	KAF1429209.1	Apolipoprotein A-I, partial	30	1	0.11
Diffuse amyloid	Heart	KAF1410998.1	Collagen alpha-1(VI) chain, partial	29	1	0.15
Diffuse amyloid	Heart	KAF1406489.1	Histidine--tRNA ligase, cytoplasmic, partial	25	1	0.07
Diffuse amyloid	Heart	KAF1415096.1	Kinesin-like protein KIF9, partial	22	1	0.04

* emPAI, exponentially modified protein abundance index.

Supplementary Table 2. Proteins detected by LC-MS/MS analysis of amyloid deposits using a protein database derived from the genome assembly (GCA_027474245) supplemented with the predicted full-length Fibrinogen Aα-chain sequence.

Amyloid morphology	Organs	Accession	Description	Score	Sequences	emPAI*
Nodular amyloid	Liver	KAM6266820**	Fibrinogen Aα-chain	471	15	0.25
Nodular amyloid	Liver	KAF1426833.1	Vitronectin, partial	310	19	0.55
Nodular amyloid	Liver	KAF1410912.1	Hemoglobin subunit beta, partial	162	5	0.75
Nodular amyloid	Liver	KAF1426285.1	Chromodomain-helicase-DNA-binding protein 3, partial	123	12	0.02
Nodular amyloid	Liver	KAF1422510.1	Trypsin, partial	107	2	0.38
Nodular amyloid	Liver	KAF1404247.1	Keratin, type I cytoskeletal 19, partial	98	4	0.27
Nodular amyloid	Liver	KAF1409665.1	Keratin, type II cytoskeletal 6A, partial	88	2	0.1
Nodular amyloid	Liver	KAF1409671.1	Keratin, type II cytoskeletal 75, partial	65	3	0.05
Nodular amyloid	Liver	KAF1398448.1	Asporin, partial	50	5	0.08
Nodular amyloid	Liver	KAF1405847.1	Bactericidal permeability-increasing protein, partial	41	1	0.07
Nodular amyloid	Liver	KAF1402331.1	Histone H2A type 2-B, partial	41	1	0.25
Nodular amyloid	Liver	KAF1428000.1	Plasminogen, partial	34	1	0.04
Nodular amyloid	Liver	KAF1417154.1	Hemoglobin subunit alpha, partial	30	3	0.48
Nodular amyloid	Liver	KAF1409669.1	Keratin, type II cytoskeletal 75, partial	30	1	0.06
Nodular amyloid	Liver	KAF1407927.1	GTP-binding protein 2, partial	29	1	0.05
Nodular amyloid	Liver	KAF1403199.1	Histone H2B 5, partial	29	2	0.24
Nodular amyloid	Liver	KAF1406489.1	Histidine--tRNA ligase, cytoplasmic, partial	28	1	0.07
Nodular amyloid	Liver	KAF1405169.1	Transcription initiation factor TFIID subunit 4, partial	26	1	0.11
Nodular amyloid	Liver	KAF1429209.1	Apolipoprotein A-I, partial	19	1	0.11
Nodular amyloid	Liver	KAF1418258.1	Phospholipid-transporting ATPase IB, partial	18	1	0.02
Nodular amyloid	Spleen	KAM6266820**	Fibrinogen Aα-chain	1469	52	0.69
Nodular amyloid	Spleen	KAF1426833.1	Vitronectin, partial	882	42	0.99
Nodular amyloid	Spleen	KAF1426285.1	Chromodomain-helicase-DNA-binding protein 3, partial	106	10	0.02
Nodular amyloid	Spleen	KAF1429209.1	Apolipoprotein A-I, partial	87	1	0.11
Nodular amyloid	Spleen	KAF1405847.1	Bactericidal permeability-increasing protein, partial	81	3	0.07
Nodular amyloid	Spleen	KAF1429220.1	Apolipoprotein A-IV, partial	78	4	0.27
Nodular amyloid	Spleen	KAF1410900.1	Hemoglobin subunit epsilon, partial	62	4	0.45

Nodular amyloid	Spleen	KAF1427924.1	Actin, alpha skeletal muscle, partial	38	2	0.08
Nodular amyloid	Spleen	KAF1417154.1	Hemoglobin subunit alpha, partial	29	1	0.22
Nodular amyloid	Spleen	KAF1419913.1	Exportin-2, partial	21	1	0.03
Nodular amyloid	Spleen	KAF1428901.1	Receptor-type tyrosine-protein phosphatase U, partial	18	1	0.02
Nodular amyloid	Spleen	KAF1415968.1	Leucine--tRNA ligase, cytoplasmic, partial	16	1	0.32
Nodular amyloid	Heart	KAM6266820**	Fibrinogen Aα-chain	563	20	0.25
Nodular amyloid	Heart	KAF1426833.1	Vitronectin, partial	175	10	0.28
Nodular amyloid	Heart	KAF1419813.1	Actin, alpha skeletal muscle B, partial	158	9	0.46
Nodular amyloid	Heart	KAF1403990.1	Actin, gamma-enteric smooth muscle, partial	133	10	0.35
Nodular amyloid	Heart	KAF1426285.1	Chromodomain-helicase-DNA-binding protein 3, partial	101	13	0.02
Nodular amyloid	Heart	KAF1415886.1	Myoglobin, partial	81	4	0.69
Nodular amyloid	Heart	KAF1428000.1	Plasminogen, partial	49	2	0.04
Nodular amyloid	Heart	KAF1412642.1	Kallikrein-14, partial	45	1	0.4
Nodular amyloid	Heart	KAF1430687.1	Collagen alpha-1(II) chain, partial	41	2	0.03
Nodular amyloid	Heart	KAF1398448.1	Asporin, partial	38	1	0.08
Nodular amyloid	Heart	KAF1397937.1	Sn1-specific diacylglycerol lipase beta, partial	35	1	0.04
Nodular amyloid	Heart	KAF1402331.1	Histone H2A type 2-B, partial	34	1	0.25
Nodular amyloid	Heart	KAF1403199.1	Histone H2B 5, partial	29	1	0.24
Nodular amyloid	Heart	KAF1396498.1	Forkhead box protein O3, partial	27	2	0.06
Nodular amyloid	Heart	KAF1414311.1	Histone H4, partial	26	1	0.3
Nodular amyloid	Heart	KAF1405169.1	Transcription initiation factor TFIID subunit 4, partial	23	1	0.11
Nodular amyloid	Heart	KAF1421293.1	DNA (cytosine-5)-methyltransferase 1, partial	20	1	0.21
Nodular amyloid	Heart	KAF1423209.1	LIM domain-binding protein 1, partial	18	1	0.07
Nodular amyloid	Heart	KAF1408707.1	Mucin-5AC, partial	13	1	0.03
Diffuse amyloid	Liver	KAF1397894.1	Actin, cytoplasmic 1, partial	220	15	0.7
Diffuse amyloid	Liver	KAF1422510.1	Trypsin, partial	153	4	0.38
Diffuse amyloid	Liver	KAF1426285.1	Chromodomain-helicase-DNA-binding protein 3, partial	110	14	0.02
Diffuse amyloid	Liver	KAF1402331.1	Histone H2A type 2-B, partial	98	4	0.56
Diffuse amyloid	Liver	KAF1429209.1	Apolipoprotein A-I, partial	82	3	0.38
Diffuse amyloid	Liver	KAF1414311.1	Histone H4, partial	81	4	0.7
Diffuse amyloid	Liver	KAF1419114.1	Annexin A5, partial	57	1	0.09
Diffuse amyloid	Liver	KAF1408665.1	Serum amyloid A protein, partial	55	2	0.24

Diffuse amyloid	Liver	KAF1410885.1	Hemoglobin subunit beta, partial	53	2	0.21
Diffuse amyloid	Liver	KAF1401624.1	histone H3.3, partial	48	2	0.22
Diffuse amyloid	Liver	KAF1398448.1	Asporin, partial	45	4	0.08
Diffuse amyloid	Liver	KAF1428000.1	Plasminogen, partial	40	1	0.04
Diffuse amyloid	Liver	KAF1403199.1	Histone H2B 5, partial	36	3	0.24
Diffuse amyloid	Liver	KAF1407490.1	Peptidyl-prolyl cis-trans isomerase A, partial	22	1	0.22
Diffuse amyloid	Liver	KAF1421293.1	DNA (cytosine-5)-methyltransferase 1, partial	20	1	0.21
Diffuse amyloid	Spleen	KAF1422510.1	Trypsin, partial	154	6	0.62
Diffuse amyloid	Spleen	KAF1426285.1	Chromodomain-helicase-DNA-binding protein 3, partial	122	13	0.02
Diffuse amyloid	Spleen	KAF1409665.1	Keratin, type II cytoskeletal 6A, partial	91	2	0.1
Diffuse amyloid	Spleen	KAF1408665.1	Serum amyloid A protein, partial	73	2	0.54
Diffuse amyloid	Spleen	KAF1404247.1	Keratin, type I cytoskeletal 19, partial	72	2	0.17
Diffuse amyloid	Spleen	KAF1428000.1	Plasminogen, partial	58	2	0.04
Diffuse amyloid	Spleen	KAF1409671.1	Keratin, type II cytoskeletal 75, partial	56	2	0.05
Diffuse amyloid	Spleen	KAF1427924.1	Actin, alpha skeletal muscle, partial	41	2	0.08
Diffuse amyloid	Spleen	KAF1402331.1	Histone H2A type 2-B, partial	39	1	0.25
Diffuse amyloid	Spleen	KAF1398448.1	Asporin, partial	37	3	0.08
Diffuse amyloid	Spleen	KAF1410885.1	Hemoglobin subunit beta, partial	34	1	0.21
Diffuse amyloid	Spleen	KAF1397809.1	Neuronal pentraxin-2, partial	32	1	0.06
Diffuse amyloid	Spleen	KAF1406489.1	Histidine--tRNA ligase, cytoplasmic, partial	27	2	0.07
Diffuse amyloid	Spleen	KAF1403199.1	Histone H2B 5, partial	26	1	0.24
Diffuse amyloid	Spleen	KAF1428901.1	Receptor-type tyrosine-protein phosphatase U, partial	23	1	0.02
Diffuse amyloid	Spleen	KAF1405169.1	Transcription initiation factor TFIID subunit 4, partial	23	1	0.11
Diffuse amyloid	Spleen	KAF1396583.1	Activating signal cointegrator 1 complex subunit 3, partial	19	1	0.01
Diffuse amyloid	Spleen	KAF1426833.1	Vitronectin, partial	17	1	0.06
Diffuse amyloid	Spleen	KAF1399997.1	NADPH oxidase 1, partial	14	2	0.05
Diffuse amyloid	Heart	KAF1408665.1	Serum amyloid A protein, partial	363	13	1.37
Diffuse amyloid	Heart	KAF1427924.1	Actin, alpha skeletal muscle, partial	314	16	0.57
Diffuse amyloid	Heart	KAF1419813.1	Actin, alpha skeletal muscle B, partial	274	14	0.57
Diffuse amyloid	Heart	KAF1409285.1	Myosin-6, partial	281	8	0.09
Diffuse amyloid	Heart	KAF1426833.1	Vitronectin, partial	171	9	0.37
Diffuse amyloid	Heart	KAF1415886.1	Myoglobin, partial	132	9	1.01

Diffuse amyloid	Heart	KAF1422510.1	Trypsin, partial	129	4	0.38
Diffuse amyloid	Heart	KAF1426285.1	Chromodomain-helicase-DNA-binding protein 3, partial	101	9	0.02
Diffuse amyloid	Heart	KAF1412736.1	Tropomyosin beta chain, partial	62	2	0.1
Diffuse amyloid	Heart	KAF1410900.1	Hemoglobin subunit epsilon, partial	44	2	0.21
Diffuse amyloid	Heart	KAF1398448.1	Asporin, partial	41	3	0.08
Diffuse amyloid	Heart	KAF1405625.1	Desmin, partial	40	2	0.14
Diffuse amyloid	Heart	KAF1419812.1	Cysteine and glycine-rich protein 3, partial	39	1	0.16
Diffuse amyloid	Heart	KAF1428000.1	Plasminogen, partial	37	1	0.04
Diffuse amyloid	Heart	KAF1415968.1	Leucine--tRNA ligase, cytoplasmic, partial	32	2	0.32
Diffuse amyloid	Heart	KAF1428045.1	Mucin-5B, partial	32	1	0.01
Diffuse amyloid	Heart	KAF1417154.1	Hemoglobin subunit alpha, partial	31	2	0.22
Diffuse amyloid	Heart	KAF1429209.1	Apolipoprotein A-I, partial	30	1	0.11
Diffuse amyloid	Heart	KAF1410998.1	Collagen alpha-1(VI) chain, partial	29	1	0.15
Diffuse amyloid	Heart	KAF1406489.1	Histidine--tRNA ligase, cytoplasmic, partial	25	1	0.07
Diffuse amyloid	Heart	KAF1415096.1	Kinesin-like protein KIF9, partial	22	1	0.04

* emPAI, exponentially modified protein abundance index.

** The amino acid sequence used for the database search was independently predicted from the Humboldt penguin genome and was identical to the subsequently deposited sequence indicated by the accession number.

Supplementary Table 3. Detected peptides of the amyloid precursor proteins by mass spectrometry**.**

Amyloid morphology	Organ	Accession	Protein name	Start	End	Score	Expect	Peptide sequence
Nodular amyloid	Liver	KAM6266820*	Fibrinogen Aα-chain	294	301	24.89	0.1	LVTPTHGR
Nodular amyloid	Liver	KAM6266820*	Fibrinogen Aα-chain	294	301	19.91	0.32	LVTPTHGR
Nodular amyloid	Liver	KAM6266820*	Fibrinogen Aα-chain	294	301	6.41	1.2	LVTPTHGR
Nodular amyloid	Liver	KAM6266820*	Fibrinogen Aα-chain	325	333	26.95	0.016	FVTGPDGPR
Nodular amyloid	Liver	KAM6266820*	Fibrinogen Aα-chain	325	333	33.11	0.0052	FVTGPDGPR
Nodular amyloid	Liver	KAM6266820*	Fibrinogen Aα-chain	257	264	34.29	0.021	QMQVVLER
Nodular amyloid	Liver	KAM6266820*	Fibrinogen Aα-chain	257	264	42.65	0.0029	QMQVVLER
Nodular amyloid	Liver	KAM6266820*	Fibrinogen Aα-chain	386	396	24.49	0.024	FTPDSPSTASR
Nodular amyloid	Liver	KAM6266820*	Fibrinogen Aα-chain	386	396	33.94	0.011	FTPDSPSTASR
Nodular amyloid	Liver	KAM6266820*	Fibrinogen Aα-chain	340	359	86.4	7.9e-009	MISSDGSDCSYLQGAGSVGR
Nodular amyloid	Liver	KAM6266820*	Fibrinogen Aα-chain	340	359	47.08	9.7e-005	MISSDGSDCSYLQGAGSVGR
Nodular amyloid	Liver	KAM6266820*	Fibrinogen Aα-chain	340	359	45.62	5.5e-005	MISSDGSDCSYLQGAGSVGR
Nodular amyloid	Liver	KAM6266820*	Fibrinogen Aα-chain	340	359	105.88	1.7e-010	MISSDGSDCSYLQGAGSVGR
Nodular amyloid	Liver	KAM6266820*	Fibrinogen Aα-chain	340	359	107.35	8.6e-011	MISSDGSDCSYLQGAGSVGR
Nodular amyloid	Liver	KAM6266820*	Fibrinogen Aα-chain	340	359	54.46	7.8e-006	MISSDGSDCSYLQGAGSVGR
Nodular amyloid	Liver	KAM6266820*	Fibrinogen Aα-chain	340	359	33.11	0.00093	MISSDGSDCSYLQGAGSVGR
Nodular amyloid	Liver	KAM6266820*	Fibrinogen Aα-chain	360	377	40.45	0.00022	EGSMYHVGGIDSFHDVEK
Nodular amyloid	Liver	KAM6266820*	Fibrinogen Aα-chain	360	377	44.89	9e-005	EGSMYHVGGIDSFHDVEK
Nodular amyloid	Spleen	KAM6266820*	Fibrinogen Aα-chain	334	339	13.06	0.35	EEVVEK
Nodular amyloid	Spleen	KAM6266820*	Fibrinogen Aα-chain	294	301	18.23	0.47	LVTPTHGR
Nodular amyloid	Spleen	KAM6266820*	Fibrinogen Aα-chain	294	301	24.29	0.12	LVTPTHGR
Nodular amyloid	Spleen	KAM6266820*	Fibrinogen Aα-chain	294	301	33.19	0.015	LVTPTHGR
Nodular amyloid	Spleen	KAM6266820*	Fibrinogen Aα-chain	294	301	21.16	0.24	LVTPTHGR
Nodular amyloid	Spleen	KAM6266820*	Fibrinogen Aα-chain	294	301	12.2	0.95	LVTPTHGR
Nodular amyloid	Spleen	KAM6266820*	Fibrinogen Aα-chain	325	333	35.28	0.0042	FVTGPDGPR
Nodular amyloid	Spleen	KAM6266820*	Fibrinogen Aα-chain	325	333	42.26	0.0018	FVTGPDGPR
Nodular amyloid	Spleen	KAM6266820*	Fibrinogen Aα-chain	325	333	20.86	0.053	FVTGPDGPR

Nodular amyloid	Spleen	KAM6266820*	Fibrinogen Aα-chain	325	333	21.88	0.084	FVTGPDGPR
Nodular amyloid	Spleen	KAM6266820*	Fibrinogen Aα-chain	325	333	21.67	0.11	FVTGPDGPR
Nodular amyloid	Spleen	KAM6266820*	Fibrinogen Aα-chain	257	264	38.41	0.0049	QMQVVLER
Nodular amyloid	Spleen	KAM6266820*	Fibrinogen Aα-chain	257	264	49.64	0.00037	QMQVVLER
Nodular amyloid	Spleen	KAM6266820*	Fibrinogen Aα-chain	257	264	40.52	0.003	QMQVVLER
Nodular amyloid	Spleen	KAM6266820*	Fibrinogen Aα-chain	257	264	41.47	0.0024	QMQVVLER
Nodular amyloid	Spleen	KAM6266820*	Fibrinogen Aα-chain	257	264	53.39	0.00016	QMQVVLER
Nodular amyloid	Spleen	KAM6266820*	Fibrinogen Aα-chain	257	264	5.45	3.5	QMQVVLER
Nodular amyloid	Spleen	KAM6266820*	Fibrinogen Aα-chain	257	264	14.28	1.4	QMQVVLER
Nodular amyloid	Spleen	KAM6266820*	Fibrinogen Aα-chain	257	264	40.11	0.005	QMQVVLER
Nodular amyloid	Spleen	KAM6266820*	Fibrinogen Aα-chain	257	264	28.07	0.079	QMQVVLER
Nodular amyloid	Spleen	KAM6266820*	Fibrinogen Aα-chain	257	264	33.13	0.025	QMQVVLER
Nodular amyloid	Spleen	KAM6266820*	Fibrinogen Aα-chain	257	264	43.72	0.0021	QMQVVLER
Nodular amyloid	Spleen	KAM6266820*	Fibrinogen Aα-chain	274	283	22.9	0.03	GDSLYHMAES
Nodular amyloid	Spleen	KAM6266820*	Fibrinogen Aα-chain	274	283	27.97	0.0041	GDSLYHMAES
Nodular amyloid	Spleen	KAM6266820*	Fibrinogen Aα-chain	386	396	27.7	0.01	FTPDSPSTASR
Nodular amyloid	Spleen	KAM6266820*	Fibrinogen Aα-chain	386	396	26.31	0.028	FTPDSPSTASR
Nodular amyloid	Spleen	KAM6266820*	Fibrinogen Aα-chain	386	396	16.04	0.22	FTPDSPSTASR
Nodular amyloid	Spleen	KAM6266820*	Fibrinogen Aα-chain	386	396	22.5	0.1	FTPDSPSTASR
Nodular amyloid	Spleen	KAM6266820*	Fibrinogen Aα-chain	386	396	14.37	0.46	FTPDSPSTASR
Nodular amyloid	Spleen	KAM6266820*	Fibrinogen Aα-chain	364	377	5.38	1.1	YHVGGIDSFHDVEK
Nodular amyloid	Spleen	KAM6266820*	Fibrinogen Aα-chain	364	377	4.21	0.4	YHVGGIDSFHDVEK
Nodular amyloid	Spleen	KAM6266820*	Fibrinogen Aα-chain	325	339	10.81	0.6	FVTGPDGPREEVVEK
Nodular amyloid	Spleen	KAM6266820*	Fibrinogen Aα-chain	325	339	13	0.38	FVTGPDGPREEVVEK
Nodular amyloid	Spleen	KAM6266820*	Fibrinogen Aα-chain	325	339	12.45	0.75	FVTGPDGPREEVVEK
Nodular amyloid	Spleen	KAM6266820*	Fibrinogen Aα-chain	325	339	28.78	0.02	FVTGPDGPREEVVEK
Nodular amyloid	Spleen	KAM6266820*	Fibrinogen Aα-chain	242	256	51.71	0.00018	SLPEMQALNIINNIR
Nodular amyloid	Spleen	KAM6266820*	Fibrinogen Aα-chain	242	256	42.32	0.0015	SLPEMQALNIINNIR
Nodular amyloid	Spleen	KAM6266820*	Fibrinogen Aα-chain	242	256	45.52	0.00073	SLPEMQALNIINNIR
Nodular amyloid	Spleen	KAM6266820*	Fibrinogen Aα-chain	242	256	47.54	0.00045	SLPEMQALNIINNIR
Nodular amyloid	Spleen	KAM6266820*	Fibrinogen Aα-chain	242	256	64.76	8.6e-006	SLPEMQALNIINNIR
Nodular amyloid	Spleen	KAM6266820*	Fibrinogen Aα-chain	242	256	35.64	0.0034	SLPEMQALNIINNIR

Nodular amyloid	Spleen	KAM6266820*	Fibrinogen Aα-chain	242	256	20.82	0.039	SLPEMQALNIINNIR
Nodular amyloid	Spleen	KAM6266820*	Fibrinogen Aα-chain	340	359	119.34	8.1e-012	MISSDGSDCSYLQGAGSVGR
Nodular amyloid	Spleen	KAM6266820*	Fibrinogen Aα-chain	340	359	92.97	1.9e-009	MISSDGSDCSYLQGAGSVGR
Nodular amyloid	Spleen	KAM6266820*	Fibrinogen Aα-chain	340	359	70.86	2.3e-007	MISSDGSDCSYLQGAGSVGR
Nodular amyloid	Spleen	KAM6266820*	Fibrinogen Aα-chain	340	359	40.69	0.00015	MISSDGSDCSYLQGAGSVGR
Nodular amyloid	Spleen	KAM6266820*	Fibrinogen Aα-chain	340	359	40.77	0.00015	MISSDGSDCSYLQGAGSVGR
Nodular amyloid	Spleen	KAM6266820*	Fibrinogen Aα-chain	340	359	51.65	4.5e-005	MISSDGSDCSYLQGAGSVGR
Nodular amyloid	Spleen	KAM6266820*	Fibrinogen Aα-chain	340	359	34.12	0.00063	MISSDGSDCSYLQGAGSVGR
Nodular amyloid	Spleen	KAM6266820*	Fibrinogen Aα-chain	340	359	73.46	1.3e-007	MISSDGSDCSYLQGAGSVGR
Nodular amyloid	Spleen	KAM6266820*	Fibrinogen Aα-chain	340	359	86.57	1.5e-008	MISSDGSDCSYLQGAGSVGR
Nodular amyloid	Spleen	KAM6266820*	Fibrinogen Aα-chain	340	359	49.71	2.2e-005	MISSDGSDCSYLQGAGSVGR
Nodular amyloid	Spleen	KAM6266820*	Fibrinogen Aα-chain	340	359	63.29	1.5e-006	MISSDGSDCSYLQGAGSVGR
Nodular amyloid	Spleen	KAM6266820*	Fibrinogen Aα-chain	340	359	64.15	9.7e-007	MISSDGSDCSYLQGAGSVGR
Nodular amyloid	Spleen	KAM6266820*	Fibrinogen Aα-chain	340	359	113.76	2.2e-011	MISSDGSDCSYLQGAGSVGR
Nodular amyloid	Spleen	KAM6266820*	Fibrinogen Aα-chain	340	359	86.93	7.1e-009	MISSDGSDCSYLQGAGSVGR
Nodular amyloid	Spleen	KAM6266820*	Fibrinogen Aα-chain	340	359	76.03	1.2e-007	MISSDGSDCSYLQGAGSVGR
Nodular amyloid	Spleen	KAM6266820*	Fibrinogen Aα-chain	360	377	1.25	1.5	EGSMYHVGGIDSFHDVEK
Nodular amyloid	Spleen	KAM6266820*	Fibrinogen Aα-chain	360	377	43.26	0.00025	EGSMYHVGGIDSFHDVEK
Nodular amyloid	Spleen	KAM6266820*	Fibrinogen Aα-chain	360	377	26.98	0.0071	EGSMYHVGGIDSFHDVEK
Nodular amyloid	Spleen	KAM6266820*	Fibrinogen Aα-chain	360	377	45.99	4.9e-005	EGSMYHVGGIDSFHDVEK
Nodular amyloid	Spleen	KAM6266820*	Fibrinogen Aα-chain	360	377	38.59	0.0007	EGSMYHVGGIDSFHDVEK
Nodular amyloid	Spleen	KAM6266820*	Fibrinogen Aα-chain	360	377	45.29	0.0001	EGSMYHVGGIDSFHDVEK
Nodular amyloid	Spleen	KAM6266820*	Fibrinogen Aα-chain	360	377	29.82	0.0026	EGSMYHVGGIDSFHDVEK
Nodular amyloid	Spleen	KAM6266820*	Fibrinogen Aα-chain	360	377	33.42	0.0022	EGSMYHVGGIDSFHDVEK
Nodular amyloid	Spleen	KAM6266820*	Fibrinogen Aα-chain	360	377	1.73	1.4	EGSMYHVGGIDSFHDVEK
Nodular amyloid	Spleen	KAM6266820*	Fibrinogen Aα-chain	360	377	6.53	0.54	EGSMYHVGGIDSFHDVEK
Nodular amyloid	Spleen	KAM6266820*	Fibrinogen Aα-chain	360	377	44.09	0.00022	EGSMYHVGGIDSFHDVEK
Nodular amyloid	Spleen	KAM6266820*	Fibrinogen Aα-chain	360	377	45.99	0.00031	EGSMYHVGGIDSFHDVEK
Nodular amyloid	Spleen	KAM6266820*	Fibrinogen Aα-chain	360	377	36.06	0.0018	EGSMYHVGGIDSFHDVEK
Nodular amyloid	Spleen	KAM6266820*	Fibrinogen Aα-chain	378	396	57.97	5.5e-005	LIPGLNPFFTPDSPSTASR
Nodular amyloid	Spleen	KAM6266820*	Fibrinogen Aα-chain	378	396	86.52	4.4e-008	LIPGLNPFFTPDSPSTASR
Nodular amyloid	Spleen	KAM6266820*	Fibrinogen Aα-chain	378	396	50.36	7.4e-005	LIPGLNPFFTPDSPSTASR

Nodular amyloid	Spleen	KAM6266820*	Fibrinogen Aα-chain	378	396	46.46	8.8e-005	LIPGLNPFFTPDSPSTASR
Nodular amyloid	Heart	KAM6266820*	Fibrinogen Aα-chain	294	301	24.2	0.12	LVTPTHGR
Nodular amyloid	Heart	KAM6266820*	Fibrinogen Aα-chain	294	301	12.89	0.63	LVTPTHGR
Nodular amyloid	Heart	KAM6266820*	Fibrinogen Aα-chain	325	333	27.58	0.011	FVTGPDGPR
Nodular amyloid	Heart	KAM6266820*	Fibrinogen Aα-chain	325	333	22.58	0.043	FVTGPDGPR
Nodular amyloid	Heart	KAM6266820*	Fibrinogen Aα-chain	257	264	39.35	0.004	QMQVVLER
Nodular amyloid	Heart	KAM6266820*	Fibrinogen Aα-chain	257	264	31.12	0.026	QMQVVLER
Nodular amyloid	Heart	KAM6266820*	Fibrinogen Aα-chain	257	264	54.86	0.00011	QMQVVLER
Nodular amyloid	Heart	KAM6266820*	Fibrinogen Aα-chain	257	264	35.92	0.0087	QMQVVLER
Nodular amyloid	Heart	KAM6266820*	Fibrinogen Aα-chain	257	264	19.41	0.14	QMQVVLER
Nodular amyloid	Heart	KAM6266820*	Fibrinogen Aα-chain	274	283	16.95	0.029	GDSLYHMAES
Nodular amyloid	Heart	KAM6266820*	Fibrinogen Aα-chain	386	396	17.85	0.053	FTPDSPSTASR
Nodular amyloid	Heart	KAM6266820*	Fibrinogen Aα-chain	386	396	15.31	0.36	FTPDSPSTASR
Nodular amyloid	Heart	KAM6266820*	Fibrinogen Aα-chain	340	359	60.31	4.40E-06	MISSDGSDCSYLQGAGSVGR
Nodular amyloid	Heart	KAM6266820*	Fibrinogen Aα-chain	340	359	47.3	5.90E-05	MISSDGSDCSYLQGAGSVGR
Nodular amyloid	Heart	KAM6266820*	Fibrinogen Aα-chain	340	359	51.23	1.60E-05	MISSDGSDCSYLQGAGSVGR
Nodular amyloid	Heart	KAM6266820*	Fibrinogen Aα-chain	340	359	20.53	0.012	MISSDGSDCSYLQGAGSVGR
Nodular amyloid	Heart	KAM6266820*	Fibrinogen Aα-chain	340	359	31.45	0.0011	MISSDGSDCSYLQGAGSVGR
Nodular amyloid	Heart	KAM6266820*	Fibrinogen Aα-chain	340	359	104.04	2.50E-10	MISSDGSDCSYLQGAGSVGR
Nodular amyloid	Heart	KAM6266820*	Fibrinogen Aα-chain	340	359	77.86	5.00E-08	MISSDGSDCSYLQGAGSVGR
Nodular amyloid	Heart	KAM6266820*	Fibrinogen Aα-chain	340	359	100.52	5.00E-10	MISSDGSDCSYLQGAGSVGR
Nodular amyloid	Heart	KAM6266820*	Fibrinogen Aα-chain	340	359	82.51	2.30E-08	MISSDGSDCSYLQGAGSVGR
Nodular amyloid	Heart	KAM6266820*	Fibrinogen Aα-chain	360	377	47.85	0.00019	EGSMYHVGGIDSFHDVEK
Nodular amyloid	Heart	KAM6266820*	Fibrinogen Aα-chain	360	377	32.69	0.003	EGSMYHVGGIDSFHDVEK
Nodular amyloid	Heart	KAM6266820*	Fibrinogen Aα-chain	360	377	31.36	0.0051	EGSMYHVGGIDSFHDVEK
Nodular amyloid	Heart	KAM6266820*	Fibrinogen Aα-chain	360	377	3.68	1	EGSMYHVGGIDSFHDVEK
Nodular amyloid	Heart	KAM6266820*	Fibrinogen Aα-chain	360	377	2.74	2.5	EGSMYHVGGIDSFHDVEK
Nodular amyloid	Heart	KAM6266820*	Fibrinogen Aα-chain	360	377	35.56	0.0014	EGSMYHVGGIDSFHDVEK
Nodular amyloid	Spleen	KAF1408665.1	Serum amyloid A protein	71	79	47.74	0.00011	GPGGAWAAK
Nodular amyloid	Spleen	KAF1408665.1	Serum amyloid A protein	19	25	25.98	0.1	DVPFLTR
Nodular amyloid	Spleen	KAF1408665.1	Serum amyloid A protein	88	95	20.63	0.07	WQSSVSGR
Nodular amyloid	Spleen	KAF1408665.1	Serum amyloid A protein	88	95	10.19	1.2	WQSSVSGR

Nodular amyloid	Spleen	KAF1408665.1	Serum amyloid A protein	88	95	20.86	0.097	WQSSVSGR
Nodular amyloid	Spleen	KAF1408665.1	Serum amyloid A protein	96	111	44.57	0.00012	GAEDTQADQEANLWGR
Nodular amyloid	Heart	KAF1408665.1	Serum amyloid A protein	21	25	22.37	0.13	PFLTR
Nodular amyloid	Heart	KAF1408665.1	Serum amyloid A protein	71	79	46.15	0.00023	GPGGAWAAK
Nodular amyloid	Heart	KAF1408665.1	Serum amyloid A protein	71	79	46.77	0.00039	GPGGAWAAK
Nodular amyloid	Heart	KAF1408665.1	Serum amyloid A protein	19	25	23.44	0.18	DVPFLTR
Nodular amyloid	Heart	KAF1408665.1	Serum amyloid A protein	19	25	23.3	0.085	DVPFLTR
Nodular amyloid	Heart	KAF1408665.1	Serum amyloid A protein	63	70	31.24	0.014	GNYDAAQR
Nodular amyloid	Heart	KAF1408665.1	Serum amyloid A protein	63	70	20.18	0.13	GNYDAAQR
Nodular amyloid	Heart	KAF1408665.1	Serum amyloid A protein	63	70	26.98	0.046	GNYDAAQR
Nodular amyloid	Heart	KAF1408665.1	Serum amyloid A protein	63	70	20.01	0.23	GNYDAAQR
Nodular amyloid	Heart	KAF1408665.1	Serum amyloid A protein	88	95	15.01	0.14	WQSSVSGR
Nodular amyloid	Heart	KAF1408665.1	Serum amyloid A protein	88	95	27.39	0.051	WQSSVSGR
Nodular amyloid	Heart	KAF1408665.1	Serum amyloid A protein	88	95	36	0.008	WQSSVSGR
Nodular amyloid	Heart	KAF1408665.1	Serum amyloid A protein	88	95	28.86	0.033	WQSSVSGR
Nodular amyloid	Heart	KAF1408665.1	Serum amyloid A protein	88	95	31.77	0.014	WQSSVSGR
Nodular amyloid	Heart	KAF1408665.1	Serum amyloid A protein	96	111	56.53	5e-006	GAEDTQADQEANLWGR
Nodular amyloid	Heart	KAF1408665.1	Serum amyloid A protein	96	111	73.59	2.5e-007	GAEDTQADQEANLWGR
Nodular amyloid	Heart	KAF1408665.1	Serum amyloid A protein	96	111	63.76	1.6e-006	GAEDTQADQEANLWGR
Nodular amyloid	Heart	KAF1408665.1	Serum amyloid A protein	96	111	86.25	3.5e-008	GAEDTQADQEANLWGR
Nodular amyloid	Heart	KAF1408665.1	Serum amyloid A protein	96	111	91.85	9.7e-009	GAEDTQADQEANLWGR

* The amino acid sequence used for the database search was independently predicted from the Humboldt penguin genome and was identical to the subsequently deposited sequence indicated by the accession number.
